# Verification of Thai ethnobotanical medicine “Kamlang Suea Khrong” driven by multiplex PCR and powerful TLC techniques

**DOI:** 10.1371/journal.pone.0257243

**Published:** 2021-09-17

**Authors:** Suthira Yanaso, Ampai Phrutivorapongkul, Darunee Hongwiset, Sirivipa Piyamongkol, Aekkhaluck Intharuksa

**Affiliations:** 1 Department of Pharmaceutical Sciences, Faculty of Pharmacy, Chiang Mai University, Mueang, Chiang Mai, Thailand; 2 Department of Pharmaceutical Chemistry and Pharmacognosy, Faculty of Pharmaceutical Sciences, Huachiew Chalermprakiet University, Bang Phli, Samutprakan, Thailand; Tallinn University of Technology, ESTONIA

## Abstract

Kamlang Suea Khrong (KSK) crude drug, a traditional Thai medicine used for oral tonic and analgesic purposes, is obtained from three origins: the inner stem bark of *Betula alnoides* (BA) or the stems of *Strychnos axillaris* (SA) or *Ziziphus attopensis* (ZA). According to the previous reports, SA contains strychnine-type alkaloids that probably cause poisoning; however, only organoleptic approaches are insufficient to differentiate SA from the other plant materials. To ensure the botanical origin of KSK crude drug, powerful and reliable tools are desperately needed. Therefore, molecular and chemical identification methods, DNA barcoding and thin-layer chromatography (TLC), were investigated. Reference databases, i.e., the ITS region and phytochemical profile of the authentic plant species, were conducted. In case of molecular analysis, multiplex polymerase chain reaction (PCR) based on species-specific primers was applied. Regarding species-specific primers designation, the suitability of three candidate barcode regions (ITS, ITS1, and ITS2) was evaluated by genetic distance using K2P model. ITS2 presented the highest interspecific variability was verified its discrimination power by tree topology. Accordingly, ITS2 was used to create primers that successfully specified plant species of authentic samples. For chemical analysis, TLC with toluene:ethyl acetate:ammonia (1:9:0.025) and hierarchical clustering were operated to identify the authentic crude drugs. The developed multiplex PCR and TLC methods were then applied to identify five commercial KSK crude drugs (CK1-CK5). Both methods correspondingly indicated that CK1-CK2 and CK3-CK5 were originated from BA and ZA, respectively. Molecular and chemical approaches are convenient and effective identification methods that can be performed for the routine quality-control of the KSK crude drugs for consumer reliance. According to chemical analysis, the results indicated BA, SA, and ZA have distinct chemical profiles, leading to differences in pharmacological activities. Consequently, further scientific investigations are required to ensure the quality and safety of Thai ethnobotanical medicine known as KSK.

## Introduction

In recent decades, there has been a revival of interest in traditional medicine since it is available, accessible, affordable, and acceptable to the local population [1]. However, taking traditional medicines cause efficacy and safety concerns due to poor quality and adulterated or counterfeit products. Accordingly, WHO strategies intend to ensure the quality, safety, proper use, and effectiveness of traditional medicines [2]. In general, medicinal herbs have been traded as crude drugs in the form of dried slices or powders of the parts used, such as the leaves, bark, and roots; therefore, identification using only organoleptic methods is impossible. Moreover, their vernacular names are often homonymic, i.e., local names refer to multiple scientific plant species. For example, *Curcuma* spp., *Rinorea* spp., *Thunbergia laurifolia*, and *Crotalaria spectabilis* are known in Thailand as “Rang Chuet”. However, *C*. *spectabilis* should be avoided because it contains poisonous pyrrolizidine alkaloids, which cause hepatotoxicity in mammals [3]. Although several medicinal herbs on the market have the same vernacular name and traditional uses, their phytochemical constituents, pharmacological activities, and toxicities might differ. Hence, reliable plant identification of commercial crude drugs is required to ensure quality and safety. The focus of this study was on “Kamlang Suea Khrong” (KSK), a homonymic Thai medicinal herb that means “the power of tiger”.

KSK, a Thai ethnobotanical medicine, refers to at least three medicinal plants, i.e., *Betula alnoides* Buch.-Ham. ex D.Don (Betulaceae), *Strychnos axillaris* Colebr. (Strychnaceae), *and Ziziphus attopensis* Pierre (Rhamnaceae) [4–8]. *B*. *alnoides* is a tree with exfoliating aromatic stem bark native to northern Thailand [4–6]. *S*. *axillaris* is a liana distributed in northeastern Thailand [4]. While, the scandent shrub *Z*. *attopensis* is predominantly found in northern and northeastern regions of Thailand [7, 8]. Furthermore, these three plants species are mainly located in other countries, including Cambodia, China, Laos, and Myanmar [9]. KSK crude drug, a material intended for medicinal use, is obtained from the inner stem bark of *B*. *alnoides* (BA) or the stem of *S*. *axillaris* (SA) or the stem of *Z*. *attopensis* (ZA) [4–8]. KSK crude drug has been traditionally used as an herbal tonic to strengthen the body in Thailand [10]. It has also been used for centuries by hard-working farmers to boost their energy and relieve their aches and pains [11]. KSK crude drug (BA or SA or ZA) has been combined with other herbs such as stems of *Cryptolepis buchananii* Roem. & Schult. (Asclepiadaceae) and *Salacia chinensis* L. (Celastraceae) to prepare oral longevity recipes, including decoction (Ya Tom) and spirits (Ya Dong Sura) [10–12]. Ya Tom is generally prepared by boiling plant materials in water (~ 1:3 v/v) at medium heat, then simmering for 15 min in a clay pot before serving. It has been suggested to sip for one glass (250 mL) twice a day regularly [10–13]. Ya Dong Sura is typically made by soaking dried plant materials in alcohol (~ 1:1 v/v) for 15–30 days. This preparation should not be consumed more than two small cups (30 mL) once daily [10–13]. KSK has also been certified by the Thai Ministry of Health to be a composition of analgesic traditional household remedy [14]. As a result, herbal pharmaceutical products contained KSK are now allowed to be registered and marketed freely throughout Thailand. According to our survey on commercial KSK crude drugs (raw materials) on Thai herbal markets, they have been sold as a single herb or an ingredient mixed with other plant materials to formulate recipes, including Ya Tom and Ya Dong Sura (S1 Fig). Commercial KSK crude drug has also been claimed to improve sexual performance, in addition to the aforementioned properties. Because it has been recommended for long-term use, the toxicity of KSK crude drug should therefore be considered.

Only clinical toxicity studies for BA and ZA aqueous extracts have been reported. The BA extract revealed no acute toxicity in mice [15]. Similarly, the extract of ZA had neither acute nor chronic toxicity in Sprague-Dawley rats [16]. However, no clinical toxicity studies of SA have been described. Interestingly, it was reported that SA has been used in the preparation of arrow poison in the Malay peninsula [17, 18]. Moreover, it contains strychnine-type alkaloids, i.e., spermostrychnine, strychnospermine, and their deacetyl derivatives that probably induce clonic convulsions [19, 20]. These data suggest that prolonged intake of SA should be thoughtfully considered because it may cause toxicity. Since the appearances of commercial BA, SA, and ZA are similar, the identification of commercial KSK crude drugs by organoleptic analysis, which is common to persons with experience and expertise, could be misleading. Official identification methods of the origin of commercial KSK crude drugs have not previously been addressed; therefore, the development of effective methods is essential.

To provide more trustworthy and accurate identification, integrated molecular and chemical techniques, e.g., DNA barcoding and chromatography, are being applied [21]. DNA barcoding is a rapid and robust technique for species identification based on DNA sequences. It has proven to be a reliable and appropriate tool applied for identifying herbal medicines [22]. The barcode loci that can differentiate between closely related plant species such as *mat*K, *rbc*L, ITS, *trn*H-*psb*A have been used [23]. To succeed in DNA barcoding, the quality of genomic DNA, the affinity of primer for amplification, sequencing methodologies, and accurate reference library are necessary to consider [22–24]. TLC is a powerful tool that is widely adopted for qualitative and quantitative herbal analyses [25]. It is a simple, cost- and time-effective technique because various samples and also reference compounds can be analyzed simultaneously [26]. These combination methods enable accurate identification of several plant species with the same vernacular name, e.g., “Baimaoteng” (*Aristolochia mollissima* Thunb. and *Solanum lyratum* Hance) [27] and “Krai-Krue” (*Aristolochia pierrei*, *A*. *tagala*, *A*. *pothieri*, *Raphistemma pulchellum*, *Gymnopetalum integrifollum*, and *Jasminum* spp.) [28]. This study aims to develop and apply DNA barcoding and thin-layer chromatography (TLC) to afford reliable identification of BA, SA, and ZA, which share the identical Thai name as “KSK” crude drugs.

## Materials and methods

### Collection of KSK

As presented in Table 1, under the permission of Department of National Parks, Wildlife and Plant Conservation (DNPWPC) and Queen Sirikit Botanic Garden (QSBG), *Betula alnoides* Buch.-Ham. ex D.Don (Betulaceae), *Strychnos axillaris* Colebr. (Strychnaceae), and *Ziziphus attopensis* Pierre (Rhamnaceae) were collected from different locations in Thailand. These plants were authenticated by Ms. Wannaree Charoensup, a botanist at the Faculty of Pharmacy, Chiang Mai University, Thailand. The authentic plant specimens (S1 Appendix) obtained from BA1-BA13, SA1-SA8, and ZA1-ZA4 were prepared, preserved, and labeled voucher specimens in the Herbarium of Faculty of Pharmacy, Chiang Mai University. The commercial KSK crude drugs, CK1-CK5, which were used for botanical origin identification, were purchased from Thai herbal markets as informed in Table 1 and S2 Fig.

**Table 1:** List of authentic plants and commercial KSK crude drugs collected from different areas in Thailand.

Samples	Codes	Collecting dates	Collection sites in Thailand (Place, District, Province)	Voucher specimens	GenBank accession numbers
*Betula alnoides*	BA1	23 Feb 2019	Doi Chang Mub, Mae Sai, Chiang Rai	YS19-BeA6	LC509583
	BA2	17 Dec 2018	Doi Inthanon, Chom Thong, Chiang Mai	YS18-BeA2-1	LC509575
	BA3	23 Jan 2019	Doi Suthep, Mueang, Chiang Mai	YS19-BeA3-1	LC509577
	BA4	29 Apr 2019	Phu Hin Rong Kla, Nakhon Thai, Phitsanulok	YS19-BeA8-1	LC509586
	BA5	23 Jan 2019	Doi Suthep, Mueang, Chiang Mai	YS19-BeA3-4	LC509578
	BA6	17 Dec 2018	Doi Inthanon, Chom Thong, Chiang Mai	YS18-BeA2-2	LC509576
	BA7	26 Jan 2019	Umphang, Umphang, Tak	YS19-BeA4	LC509579
	BA8	5 Feb 2019	QSBG*, Mae Rim, Chiang Mai	YS19-BeA5-1	LC509580
	BA9	5 Feb 2019	QSBG*, Mae Rim, Chiang Mai	YS19-BeA5-2	LC509581
	BA10	5 Feb 2019	QSBG*, Mae Rim, Chiang Mai	YS19-BeA5-3	LC509582
	BA11	28 Apr 2019	Phu Ruea, Phu Ruea, Loei	YS19-BeA7-1	LC509584
	BA12	28 Apr 2019	Phu Ruea, Phu Ruea, Loei	YS19-BeA7-2	LC509585
	BA13	29 Apr 2019	Phu Hin Rong Kla, Nakhon Thai, Phitsanulok	YS19-BeA8-2	LC509587
*Strychnos axillaris*	SA1	20 Jan 2019	Weonbuk, Khong Chiam, Ubon Ratchathani	YS19-StA2-1	LC509589
	SA2	20 Jan 2019	Weonbuk, Khong Chiam, Ubon Ratchathani	YS19-StA2-2	LC509590
	SA3	27 Jun 2018	Faculty of Pharmacy, Ubon Ratchathani University, Warin Chamrap, Ubon Ratchathani	YS18-StA1	LC509588
	SA4	20 Jan 2019	Huay Phai, Khong Chiam, Ubon Ratchathani	YS19-StA2-3	LC509591
	SA5	21 Jan 2019	Huay Phai, Khong Chiam, Ubon Ratchathani	YS19-StA3-1	LC509592
	SA6	21 Jan 2019	Huay Phai, Khong Chiam, Ubon Ratchathani	YS19-StA3-2	LC509593
	SA7	27 Apr 2019	Phu Thok Temple, Sri Wilai, Bueng Kan	YS19-StA4-1	LC509594
	SA8	27 Apr 2019	Phu Thok Temple, Sri Wilai, Bueng Kan	YS19-StA4-2	LC509595
*Ziziphus attopensis*	ZA1	19 Sep 2018	Nasabang, Sri Wilai, Bueng Kan	YS18-ZiA1	LC509596
	ZA2	27 Apr 2019	Phu Thok Temple, Sri Wilai, Bueng Kan	YS19-ZiA3-2	LC509599
	ZA3	6 Mar 2019	Nasabang, Sri Wilai, Bueng Kan	YS19-ZiA2	LC509597
	ZA4	27 Apr 2019	Phu Thok Temple, Sri Wilai, Bueng Kan	YS19-ZiA3-1	LC509598
Commercial KSK crude drugs	CK1	17 May 2018	Warorot market, Mueang, Chiang Mai	-	-
	CK2	15 Dec 2018	Mae Tha market, Mae Tha, Lumphun	-	-
	CK3	11 Jan 2019	Herbal market 1, Sri Wilai, Bueng Kan	-	-
	CK4	6 Mar 2019	Herbal market 2, Sri Wilai, Bueng Kan	-	-
	CK5	27 Apr 2019	Herbal market 3, Sri Wilai, Bueng Kan	-	-

*QSBG = Queen Sirikit Botanic Garden.

### Molecular identification

#### Isolation of total DNA

Leaves of the authentic plants (BA1-BA13, SA1-SA8, and ZA1-ZA4) and plant materials of the commercial KSK crude drugs (CK1-CK5) were extracted using an i-genomic Plant DNA extraction mini kit (Intron biotechnology, Korea) following the manufacturer’s instructions to obtain total DNA.

#### Polymerase chain reaction (PCR) amplification and sequence analysis

Short DNA fragments from the internal transcribe spacer (ITS) region were amplified using PCR. The reaction mixture for each sample consisted of 12.5 μL of 2X PCR buffer for KOD FX Neo, 5 μL of 0.4 mM dNTP, 0.75 μL of 0.15 μM each universal primer [29], which were ITS5S (5′-CCTTATCATTTAGAGGAAGGAG-3′) and ITS4 (5′-TCCTCCGCTTATTGATATGC-3′), 100 ng of genomic plant DNA, and 0.5 μL of 0.5 units of KOD FX Neo DNA polymerase (Toyobo, Japan). Deionized water was added to a final volume of 25 μL.

PCR amplification was carried out under the following cycling parameters: initial denaturation at 94°C for 2 min followed by 30 cycles of denaturation at 94°C for 15 s, annealing at 53°C for 30 s, and elongation at 68°C for 45 s. A final elongation step was performed at 68°C for 5 min. The PCR products were visualized by gel electrophoresis using a 2.2% agarose gel. They were then subjected to purification with a MEGAquick-spin^TM^ Plus Total Fragment DNA Purification Kit (Intron Biotechnology, Korea). A purified PCR fragment was directly sequenced using an ABI PRISM 3730 XL sequencer (Applied Biosystems, USA).

#### Data analysis

**Fig 1:**
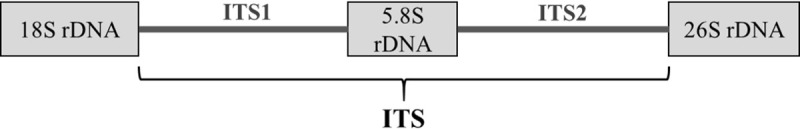
Structure of the ITS region of nuclear ribosomal DNA. The ITS region consists of the ITS1, ITS2, and 5.8S rDNA genes [30].

The obtained nucleotide sequences were examined, aligned, and manually edited using BioEdit version 7.0.5.3 and MEGA-X version 10.0.5 software. All sequences were then submitted to the GenBank database under accession number LC509575-99 (Table 1). Single nucleotide polymorphisms (SNPs) of each plant species were explored using the latter software. The discrimination power among three candidate barcodes (ITS, ITS1, and ITS2) (Fig 1) was compared using Kimura 2-parameter (K2P) distance matrices. The selected region was then tested for reliability *via* tree topology analysis based on the neighbor-joining (NJ) method with 1000 bootstrap replicates. Finally, DNA sequences from the selected barcode region were used as a reference database for species-specific primer design to identify commercial KSK crude drugs.

#### Species identification of commercial KSK crude drugs by multiplex PCR

Molecular identification of CK1-CK5 was carried out using multiplex PCR. The nucleotide sequences of the selected region of the authentic medicinal plants in this study together with other medicinal plants in the genera *Betula*, *Strychnos*, and *Ziziphus* from the GenBank database were aligned (S1 Spreadsheet) to search the different sites in order to design species-specific primers. Three sets of species-specific primers (Table 2), i.e., KSKITSF/BeAITS2R, KSKITSF/StAITS2R, and KSKITSF/ZiAITS2R, as well as an internal control primer, KSKITSF/ITS4, were utilized simultaneously in multiplex PCR amplification. *Strychnos nux-blanda* A.W. Hill (Strychnaceae), which was authenticated by the botanist and kept in the herbarium (voucher specimen no. YS19-StN1), was used as a negative control. The reaction mixture and PCR amplification conditions used were the same as those used in the aforementioned protocol. The amplicons were finally determined by 2.2% agarose gel electrophoresis and captured on a UV tray using an imager (Gel Doc^TM^ EZ, Bio-Rad Laboratories, USA) and Image Lab version 3.0 software.

**Table 2:** Primers used for multiplex PCR.

Primers	Purpose	Sequences (5’→ 3’)	Tm* (°C)
KSKITSF	5.8 rDNA region specific	TCT CGC ATC GAT GAA GAA	50.12
ITS4	26s rDNA region specific	TCC TCC GCT TAT TGA TAT GC	51.67
BeAITS2R	*Betula alnoides* specific	CCA ATT TCT GCC CCA CT	52.37
StAITS2R	*Strychnos axillaris* specific	ATC CTC TCC AGC GAC AGA	55.28
ZiAITS2R	*Ziziphus attopensis* specific	CCG GGG ACC TAC GTT TT	54.82

*Tm = melting temperature.

### Chemical identification

#### Sample extraction

The stem bark of BA1-BA5, stems of SA1-SA2, stems of ZA1-ZA2, and plant materials CK1-CK5 were cut into small pieces and dried in a hot air oven at 50°C for 72 h. The dried samples were ground into fine powder and extracted with 95% ethanol on a shaker (SHR-2D, Daihan Scientific, Korea). After 24 h of extraction, the samples were filtered, and the filtrates were concentrated under reduced pressure. For each sample, this extraction process was repeated three times. The crude extracts of all samples were kept in a refrigerator until use.

#### TLC condition development

TLC analysis was carried out using activated silica gel 60 F_254_ TLC plates (Merck, Germany) as the stationary phase. Betulinic acid (Chemfaces, China) and lupeol (Chemfaces, China) were utilized as standard references. Five microliters of each 1 mg/mL standard reference solution and each 15 mg/mL sample extract were loaded with a 4 mm bandwidth on TLC plates using a sample applicator (Linomat V, Camag, Switzerland) equipped with a 100 μL syringe. Then the plates were developed in the optimized mobile phase to a distance of 80 mm. After that, the dried plates were sprayed with anisaldehyde-sulfuric acid reagent before drying in an oven at 105°C for 20 min. The TLC chromatograms were viewed and photo-recorded under UV light at 366 nm using a TLC viewer (TLC visualizer 2, Camag, Switzerland). The results from this method were orderly rearranged using visionCATS version 2.3 software.

#### Data analysis

Hierarchical cluster analysis (HCA), a simple and rapid clustering method, was applied for qualitative analysis to differentiate the three plant species. The TLC data were used to generate row and column data matrices. The data consisted of sample codes as rows, R_f_ values as columns, and the number 0 (absent) and 1 (available) as the input variables (S2 Appendix). These data were clustered as a dendrogram using R Studio version 1.2.5033 software.

#### Species identification of commercial KSK crude drugs by TLC

Chemical identification of CK1-CK5 was analyzed using TLC chromatograms and HCA. The authentic samples of each plant species, which showed the clearest chromatograms in the prior experiment, were chosen as references for the species identification of CK1-CK5.

## Results

### Generation of the DNA barcode database and DNA nucleotide analysis

DNA sequences in the ITS region obtained from the leaf extracts of the authentic plants (BA1-BA13, SA1-SA8, and ZA1-ZA4) were chosen as the DNA barcode database for KSK in this study (S2 Spreadsheet). They were submitted to the GenBank database under accession number LC509575-99 (Table 1). The lengths of the ITS regions from *B*. *alnoides*, *S*. *axillaris* and, *Z*. *attopensis* were 601 bp, 616–617 bp, and 645 bp, respectively. Moreover, there was no intraspecific variation for *B*. *alnoides*, as BA1-BA13 shared the same haplotype. Additionally, the DNA sequences of *S*. *axillaris* and *Z*. *attopensis* demonstrated single nucleotide polymorphisms (SNPs) with four haplotypes for each species (S3 Appendix). As shown in Table 3, DNA nucleotide variation of *S*. *axillaris* demonstrated two base substitutions (at nucleotide positions 138 and 180) and one base insertion/deletion (at nucleotide positions 402), whereas the DNA nucleotide variation of *Z*. *attopensis* displayed three base substitutions (at nucleotide positions 107, 510, and 611).

**Table 3:** SNPs in the ITS region amplified from the leaves of *S*. *axillaris* and *Z*. *attopensis*.

***Strychnos axillaris***		**ITS**		
**Haplotype**	**GenBank accession no.**	138	180	402
1	LC509588	G	T	-
2	LC509589-93	G	Y	-
3	LC509594	G	Y	C
4	LC509595	R	C	C
***Ziziphus attopensis***		**ITS**		
**Haplotype**	**GenBank accession no.**	107	510	611
1	LC509596	T	C	R
2	LC509597	W	Y	R
3	LC509598	T	Y	R
4	LC509599	T	Y	A

A = adenosine, C = cytosine, G = guanine, R = guanine or adenosine, T = thymine, W = adenosine or thymine, Y = thymine or cytosine,— = absence of base nucleotide.

**Fig 2:**
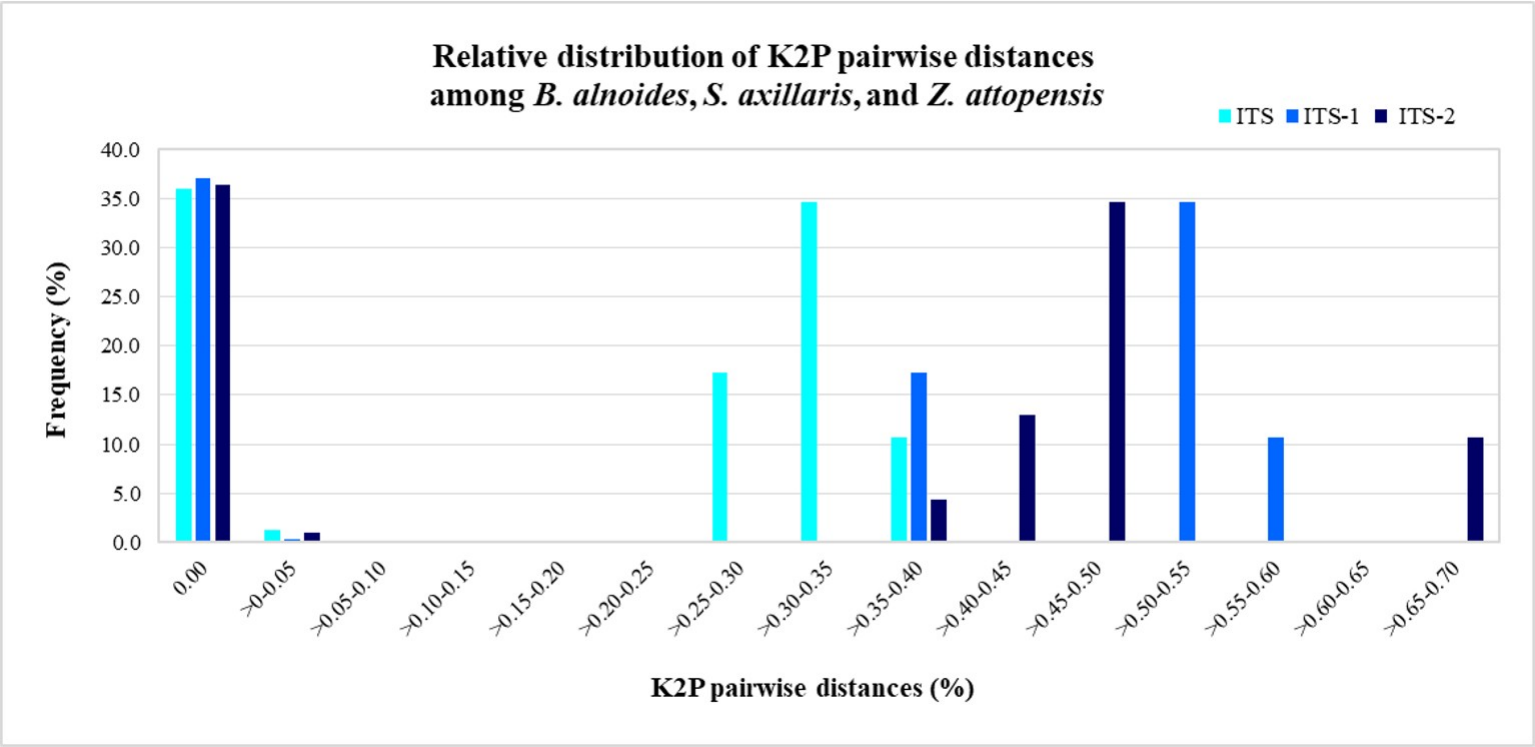
Relative distribution of the K2P pairwise distances of ITS, ITS1, and ITS2 among *B*. *alnoides*, *S*. *axillaris*, and *Z*. *attopensis*.

Since commercial KSK crude drugs are available as their dried stem bark or stems form, their DNA quality and quantity might not be sufficient for amplification. To solve this problem, species-specific primers providing short amplicons were applied. Three candidate DNA barcodes, ITS, ITS1, and ITS2, were investigated to obtain a suitable database for species-specific primer design. Table 4 demonstrates the properties of ITS, ITS1, and ITS2 region alignment from authentic samples (S3 Spreadsheet). The results showed that the ITS possessed the most extended length range and alignment range of DNA, followed by the ITS1 and then the ITS2 regions. The ITS2 region expressed the most variable sites and parsimony-informative sites, whereas ITS data indicated the most conserved sites. The discrimination power of the candidate regions was then compared using genetic distance analysis based on the K2P model (S4 Spreadsheet). The percentage of K2P pairwise distances was calculated as the relative distribution of interspecific divergence (Fig 2). The K2P pairwise distances (%) of the ITS, ITS1, and ITS2 regions among *B*. *alnoides*, *S*. *axillaris*, and *Z*. *attopensis* were 0.00–0.40, 0.00–0.60, and 0.00–0.70, respectively. These results indicated that the ITS2 region exhibited the highest variation in interspecific distances, followed by the ITS1 and ITS regions. ITS2 was therefore considered to be the most suitable DNA region used for commercial KSK crude drug identification.

**Table 4:** Properties of the ITS, ITS1, and ITS2 barcoding regions of *B*. *alnoides*, *S*. *axillaris*, and *Z*. *attopensis*.

DNA barcode regions	ITS	ITS1	ITS2
Length range (bp)	601–645	217–262	219–223
Aligned length (bp)	681	271	242
Conserve sites (n, %)	448, 65.78%	160, 59.4%	130, 53.72%
Variable sites (n, %)	233, 34.21%	111, 40.96%	112, 46.28%
Parsimony-informative sites (n, %)	231, 33.92%	110, 40.59%	111, 45.87%
Singleton sites (n, %)	1, 0.15%	1, 0.37%	1, 0.41%

**Fig 3:**
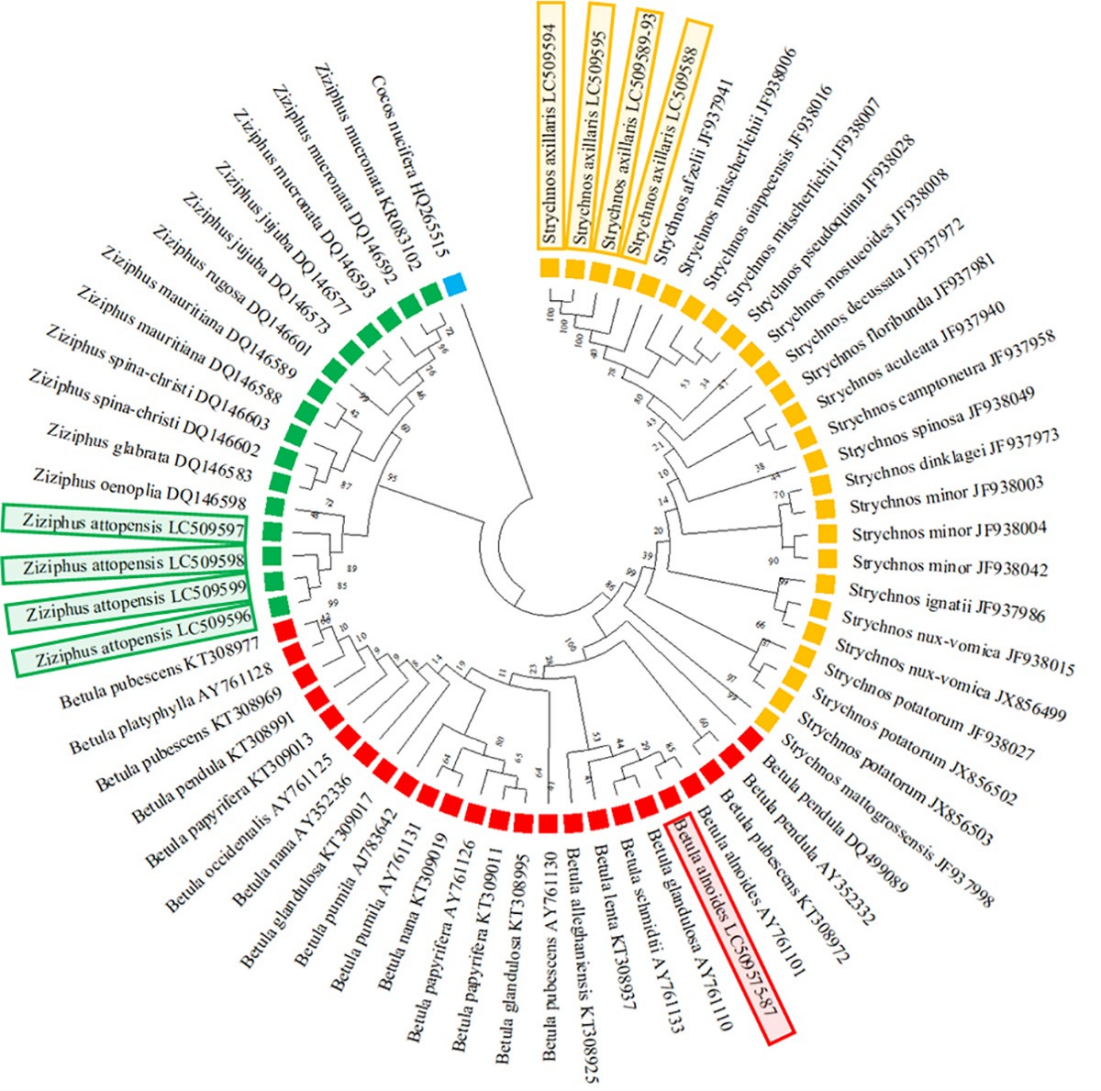
**Neighbor-joining (NJ) tree of *Betula* (red), *Strychnos* (yellow), *Ziziphus* (green) with *Cocos nucifera* (blue) as the out-group.** Red, yellow, and green boxes represent the identified species in our research. Numbers after each species refer to their GenBank accession number. A dendrogram was constructed with MEGA X version 10.0.5 software based on the aligned nucleotide sequences of the ITS2 region. The NJ bootstrap support values are shown for each branch.

Because the development of actual protocols for species identification by DNA barcoding remains ambiguous, method performance should be evaluated [31]. Thus, tree topology analysis using the neighbor-joining (NJ) method was carried out. DNA sequences at the ITS2 region of BA1-BA13, SA1-SA8, and ZA1-ZA4 of this work, as well as those of other medicinal plant species in the genera *Betula*, *Strychnos*, and *Ziziphus* registered in the GenBank database were selected as the in-groups (S1 Spreadsheet) while *Cocos nucifera* retrieved from the GenBank database (GenBank accession no. HQ265515) was employed as an out-group. The NJ tree demonstrated that the obtained DNA sequences from the ITS2 region of *B*. *alnoides* (LC509575-87), *S*. *axillaris* (LC509588-95), and *Z*. *attopensis* (LC509596-99) clustered into their own clades and were separated from the other species (Fig 3). These results suggested that their nucleotide sequences in the ITS2 region were species-specific; subsequently, they were employed as reference databases for identifying the origin of commercial KSK crude drugs.

### Chemical profiling of KSK

**Fig 4:**
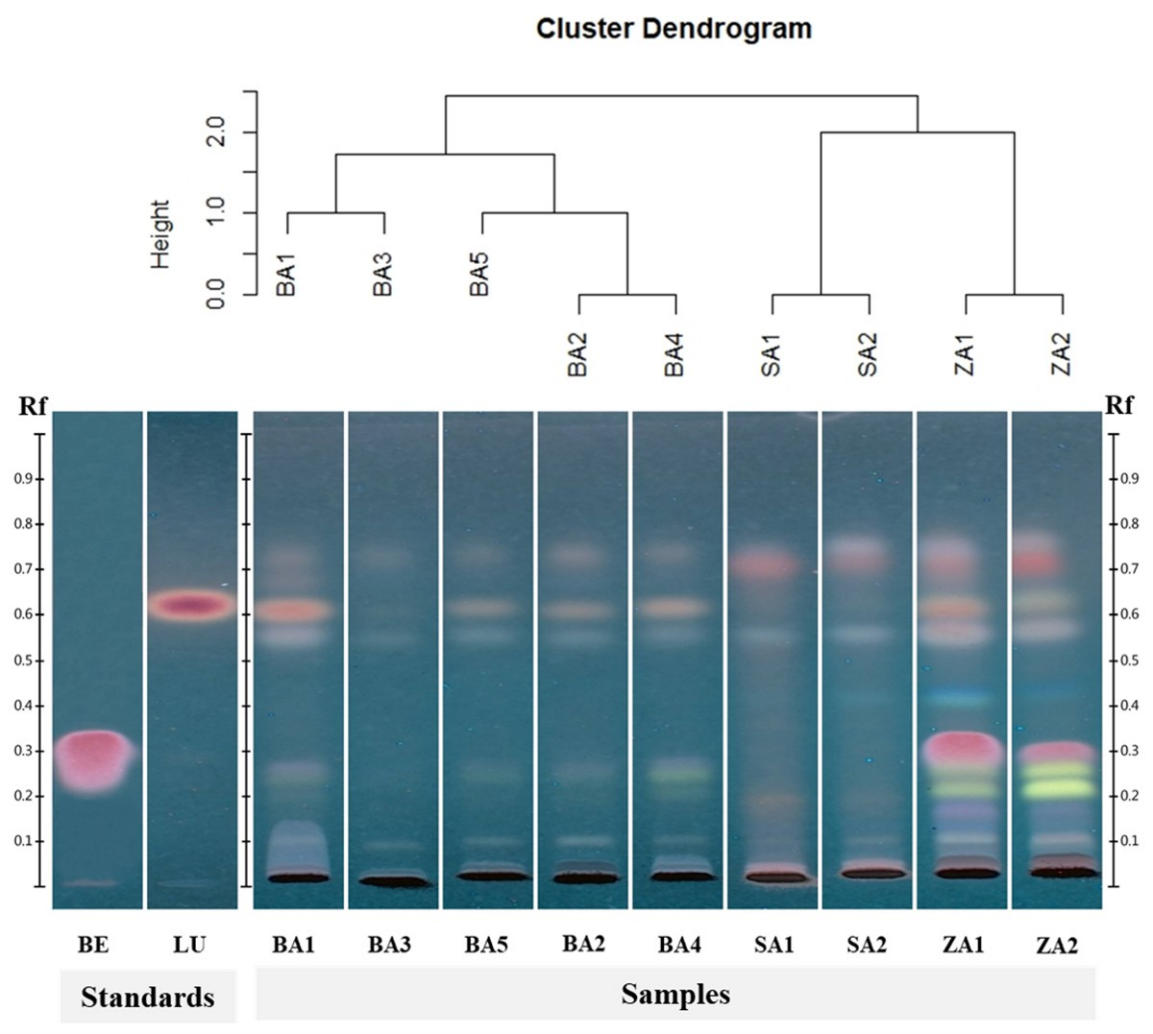
Cluster dendrogram from hierarchical cluster analysis (HCA) and TLC chromatograms under UV light at 366 nm after derivatization with anisaldehyde-sulfuric acid showing the presence of betulinic acid (BE), lupeol (LU), BA1-BA5, SA1-SA2, and ZA1-ZA2.

The mobile phase system that gave the best resolution and separation between each species was the combination of toluene:ethyl acetate:ammonia (1:9:0.025). The chromatograms (Fig 4) were visualized under UV light at 366 nm after spraying with anisaldehyde-sulfuric acid reagent. HCA was then applied to provide a rapid cluster of plant species. It was demonstrated that the cluster dendrogram, based on the R_f_ values of the components from BA1-BA5, SA1-SA2, and ZA1-ZA2 (S2 Appendix), was clearly clustered among different species (Fig 4). The TLC chromatograms of BA1, SA1, and ZA1 gave the clearest separation and resolution; therefore, they were chosen as references for identifying the origin of the commercial KSK crude drugs.

### Species identification of the commercial KSK crude drugs by multiplex PCR and TLC

In this study, CK1-CK5, which were unidentified by the organoleptic method and purchased from herbal markets, were identified by both molecular and chemical methods. The results from authentic samples, i.e., BA1, SA1, and ZA1, were used as references.

**Fig 5:**
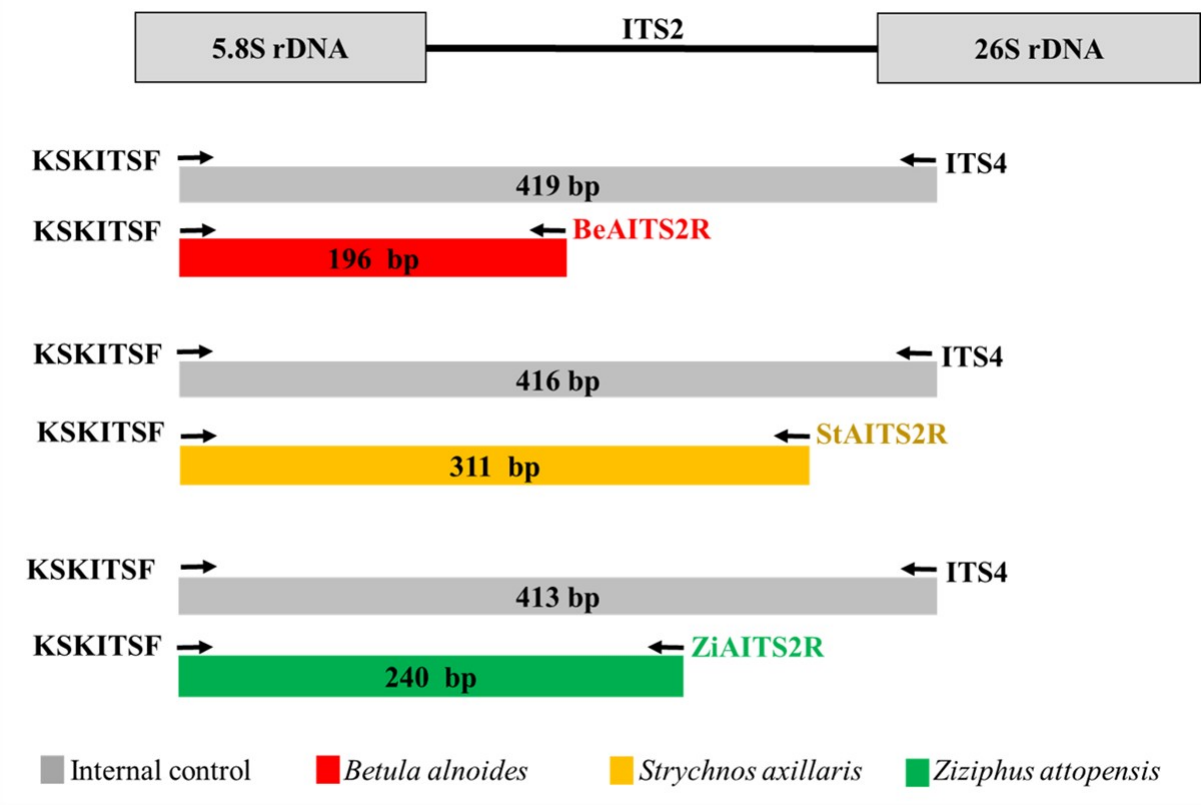
Amplification fragment sites of species-specific primer sets for the internal control, *B*. *alnoides*, *S*. *axillaris*, and *Z*. *attopensis*. The arrow indicates the orientations and approximate positions of the species-specific primers. The gray, red, yellow, and green bars represent the PCR products from multiplex PCR.

For molecular analysis, multiplex PCR, a molecular technique that amplifies DNA using species-specific primers under a single set of PCRs [32], was established. DNA nucleotides of selected region ITS2 of the authentic samples were applied for manually designing and creating species-specific primers. As presented in Fig 5, reverse species-specific primers based on the ITS2 region, i.e., BeAITS2R, StAITS2R, and ZiAITS2R, were designed for *B*. *alnoides*, *S*. *axillaris*, and *Z*. *attopensis*, respectively. Furthermore, a forward primer, KSKITSF, was designed and used to amplify the 5.8S rDNA region, while ITS4, a universal reverse primer, was also used to amplify 26S rDNA. KSKITSF and ITS4 were used as internal controls. Then, multiplex PCR, concurrently utilizing all of these primers in a single PCR, was established to identify KSK crude drugs.

**Fig 6:**
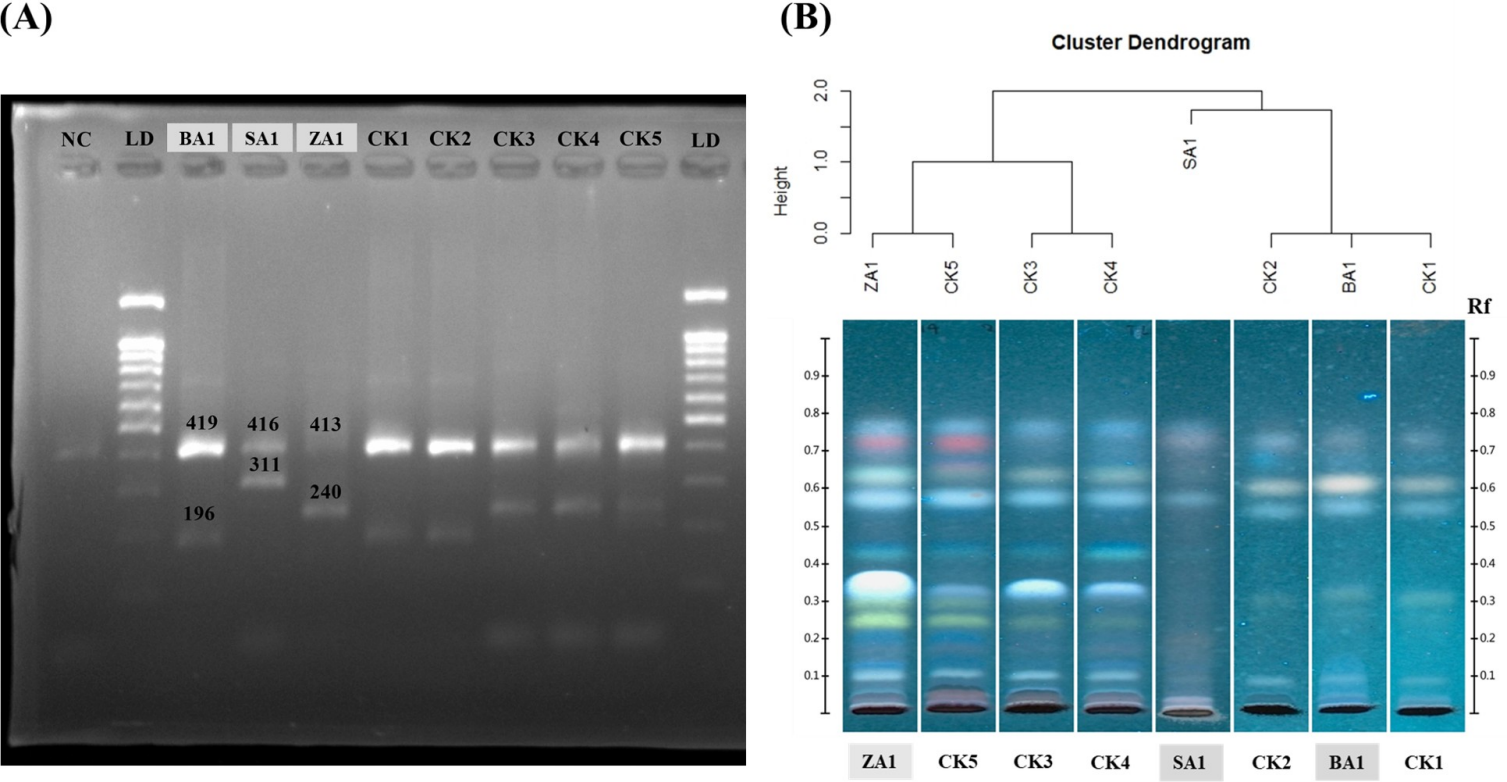
Commercial KSK crude drugs (CK1-CK5) were identified by comparison with BA1, SA1, and ZA1 in the gray blanket. (A) Image of the amplicons from multiplex PCR detected by 2.2% agarose gel electrophoreses. NC = negative control (*S*. *nux-blanda*); LD = ladder. (B) Cluster dendrogram by HCA analysis and TLC fingerprints under UV light at 366 nm after derivatization with anisaldehyde-sulfuric acid.

To verify the efficiency of species-specific primers, the selected authentic samples (BA1, SA1, and ZA1) and negative control sample (*S*. *nux-blanda*) were examined using multiplex PCR. As shown in Fig 6A and S3 Fig, the following amplicons were revealed: 419 and 196 bp of BA1, 416 and 311 bp of SA1, 413 and 240 bp of ZA1 as internal controls and species-specific amplicons, respectively. For the negative control, only the internal control amplicon was detected. These obtained results corresponding to the expected results (Fig 5) indicated that all designed primers were proper to identify CK1-CK5. The amplicons from the multiplex PCRs of CK1-CK5 in Fig 6A and S3 Fig also comprised 2 fragments, the upper and lower bands. All upper bands gave fragment sizes similar to those of the internal control. CK1-CK2 possessed a lower band corresponding to BA1, whereas CK3-CK5 provided a lower band consistent with ZA1. These results demonstrated that CK1-CK2 originated from *B*. *alnoides* and that CK3-CK5 were derived from *Z*. *attopensis*.

Chemical identification was applied to CK1-CK5 using the TLC technique followed by HCA analysis. Separation was performed using toluene:ethyl acetate:ammonia (1:9:0.025) as the mobile phase. TLC chromatograms were observed under UV light at 366 nm after derivatization with anisaldehyde-sulfuric acid. The cluster dendrogram (S2 Appendix) and TLC chromatograms in Fig 6B show that CK1-CK2 categorized as BA1 represented *B*. *alnoides* and CK3-CK5 as ZA1 represented *Z*. *attopensis*, which corresponded to the molecular analysis-based results.

## Discussion

DNA barcoding is particularly useful for differentiating closely related plant species with similar morphologies and chemical profiles. However, dried or processed materials or even plant specimens with high numbers of secondary metabolites might cause a problem in terms of the quality and quantity of DNA [33]. Although chromatography, especially TLC, is one of the official identification methods available in various herbal Pharmacopoeias, certain limitations exist because of the variation in chemical constituents in plant materials due to age, growth, and storage conditions [25, 26]. In addition, closely related species with similar chemical profiles cannot be distinguished from each other [21]. To alleviate these challenges, DNA barcoding and TLC were incorporated to identify commercial KSK crude drugs in this study. This integrated approach has also previously been applied to successfully identify and control the quality of ethnobotanical medicines, for example, *Marsdenia tenacissima* (traditional Chinese medicine) and *Galphimia glauca* (Mexican medicinal species) [34, 35].

Generally, DNA barcodes of unknown samples would typically be compared with reference DNA barcodes to find the matching species [32]. However, crude drugs derived from the bark or stems of plants are DNA-poor sources in living trees. Moreover, their DNA nucleotides are complicated to isolate and often highly degraded or sometimes contaminated with endophytic fungi [36]. These problems might affect DNA sequencing; therefore, species-specific primers were considered for certain successful identification [33]. To produce a DNA reference database of KSK, ITS, the commonly used region for DNA barcoding of several plant species, was selected [37]. With regard to this study, the ITS2 region expressed the highest discrimination power when performing genetic distance analysis using the K2P model. It was then subjected to a performance test for its reliability using tree topology analysis *via* the NJ method. The findings proved that the ITS2 region provided adequate information to identify the plant species of KSK and could distinguish between the closely related medicinal plant species. ITS2 is an ideal barcode due to its short length, ease of amplification, and highly variable discrimination of closely related species [38]. Several previous reports have indicated that the use of the ITS2 region, a mini-barcode, could successfully discriminate plants with the same vernacular name, for example, traditional Chinese medicines named Gao-ben (*Ligusticum sinense* and *L*. *jeholense*) [39] and Luoshiteng (*Trachelospermum jasminoides*, *Ficus tikoua*, *F*. *pumila*, and *Euonymus fortune* [40]. Therefore, the ITS2 region was suitable for generating species-specific primers, which were employed to identify the origin of the commercial KSK crude drugs. When designing specific primers, many aspects are taken into account including primer length, primer length difference, GC content, nucleotide composition at the primer 3′ end, melting temperature (Tm), and melting temperature difference [41]. Furthermore, the DNA analysis of closely related species was also considered [33]. Upon careful species-specific primer design, a successful multiplex PCR method to identify the botanical origins of the commercial KSK crude drugs was obtained. This technique has also been used to authenticate other medicinal plant species such as *Aralia continentalis*, *Angelica biserrata* [42]. Moreover, it has been practically applied for identification of *Atractylodes macrocephala*, *Glycyrrhiza uralensis*, and *Zingiber officinale* in ginseng decoctions [43].

The chemical profiles of the crude ethanolic extracts from the bark samples of BA1-BA5, stems of SA1-SA2, and stems of ZA1-ZA2 were obtained using TLC, a time-saving, economical and uncomplicated technique [26]. The extraction method applied in this study imitated the traditional Thai preparation “Ya Dong Sura”, which was formulated by soaking sliced or powdered medicinal plants in liquor. Therefore, the obtained chemical constituents would correspond to cultural consumption. The TLC fingerprints (Fig 4) of the authentic plant extracts (BA1-BA5, SA1-SA2, and ZA1-ZA2) were compared within the same species. Lupeol and betulinic acid were used as reference markers. The chromatographic pattern of BA3 was very weak when compared to BA1-BA5. This result indicated that BA3 demonstrated fewer chemical constituents than the other samples. This variation could be due to differences in environmental conditions and the age of the collected plants [44]. BA1-BA5 were gathered from northern areas of Thailand, at elevations ranging from 1,167 to 1,660 meters above sea level. The collected area of BA3 was different than BA1, BA2, and BA4, but similar to BA5. Meanwhile, BA3 was obtained from smaller and younger tree than others. Consequently, the difference in age of this plant species is a promising factor in the chemical component differences. These findings are in agreement with several prior investigations [45–47]. Due to the variation of the constituents, the standardization which is used to ensure the quality, including efficacy and safety, of herbal medicines is necessary. A standardization involves pre-determining one or more biochemical constituents as either active or as marker compounds [48]. The chemical markers including lupeol and betulinic acid are concentrated in this work. The comparison of chemical profiles among BA, SA, and ZA revealed that betulinic acid was the major substance in ZA, while lupeol was the main component of BA. However, betulinic acid and lupeol have also been found in other plants such as *Arbutus menziesii*, *Lycospersicon esculentum*, and *Morus alba* [49, 50]. Therefore, these chemical substances could be used as specific markers to differentiate the origin of KSK crude drug, including BA, SA, and ZA. This chemical analysis was not addressed whether these markers could discriminate KSK crude drug from other plant species. In addition, the dissimilarity of chemical substances among these three species may result in different pharmacological effects. Additional scientific studies are needed to confirm this issue.

The plant origins of five commercial KSK crude drugs (CK1-CK5) obtained from the north and northeastern Thailand as raw materials, the dried fragmented bark or stems of the individual plants, were determined. The results of both the multiplex PCR and TLC-based techniques indicated that CK1-CK2 were BA, CK3-CK5 were ZA, and none of the commercial KSK crude drugs in this experiment were SA. The concerned SA was not observed as commercial KSK crude drugs because SA may be generally used in only specific areas, or it may be preferable to collect for use rather than for sale. Because KSK crude drug has been officially accepted by the Thai Ministry of Health to be an ingredient of analgesic traditional household remedy [14], it could be found in herbal medicine products as various dosage forms including tincture, bolus, and capsule, all of which are freely sold in Thailand despite being marketed as raw materials. The developed chemical and molecular methods could also be adapted to standardize KSK in order to produce safe and effective herbal medicinal products as well.

## Conclusion

Because KSK crude drug has been recommended for long-term usage, its toxicity should be considered. To avoid the intake of SA, which may induce toxicity after prolonged use, identification of KSK crude drug is desperately needed. The findings of this study suggested simple, fast, and effective multiplex PCR and TLC chromatographic techniques that were successfully adopted for the identification of commercial KSK crude drugs distributed in Thailand. The proposed molecular and chemical identification methods could be applied for routine quality-control of KSK crude drugs for consumer reliance. Despite the fact that BA, SA, and ZA have the same name as KSK crude drug, their chemical profiles differ, potentially resulting in a wide range of pharmacological activities. Accordingly, further studies should provide insight into this issue.

## Supporting information

S1 Fig Commercial KSK crude drugs in Thai herbal markets. (PDF)

S2 Fig KSK crude drugs used in this study. (PDF)

S3 Fig The original image of the amplicons from multiplex PCR. (PDF)

S1 Appendix The authentic KSK plants collected in this study. (PDF)

S2 Appendix Input data for HCA cluster analysis. (PDF)

S3 Appendix Variation in ITS region of *S. axillaris* and *Z. attopensis*. (PDF)

S1 Spreadsheet ITS2 sequence alignment for species-specific primers design and tree topology analysis. (XLSX)

S2 Spreadsheet ITS sequence of *B. alnoides*, *S. axillaris*, and *Z. attopensis* in this study. (XLSX)

S3 Spreadsheet ITS, ITS1, and ITS2 sequence alignment of the authentic samples. (XLSX)

S4 Spreadsheet K2P distance analysis based on K2P model. (XLSX)
