# Supplementary material for: Verification of Thai ethnobotanical medicine “Kamlang Suea Khrong” driven by multiplex PCR and powerful TLC techniques
Source: PLoS One. 2021 Sep 17;16(9):e0257243. doi: 10.1371/journal.pone.0257243 (PMC8448358; doi:10.1371/journal.pone.0257243)
Supplement: S1 Fig — (PDF) [file pone.0257243.s001.pdf]

A

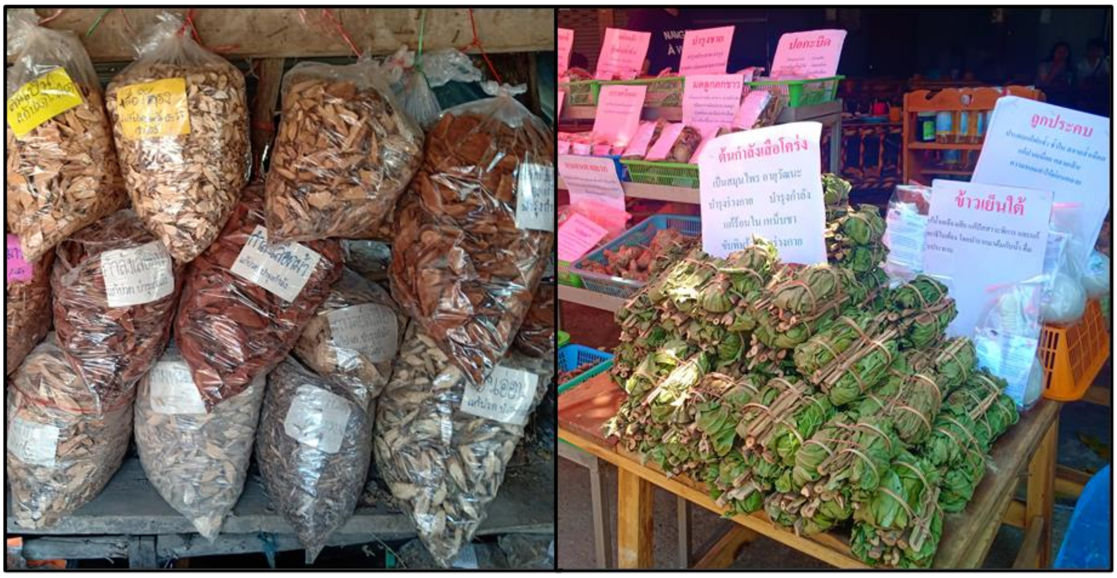


B

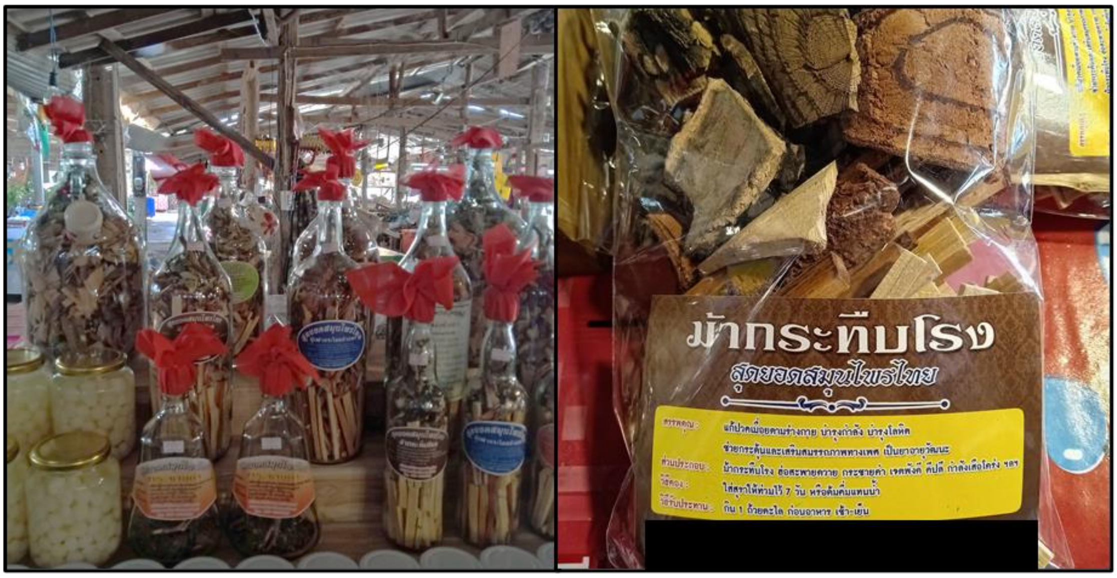


**S1 Fig. Commercial KSK crude drugs in Thai herbal markets.** They were sold as single herb (A) or admixture in “Ya tom” or “Ya dong” recipes (B)**.**
