# Supplementary figures and images for: Verification of Thai ethnobotanical medicine “Kamlang Suea Khrong” driven by multiplex PCR and powerful TLC techniques
Source: PLoS One. 2021 Sep 17;16(9):e0257243. doi: 10.1371/journal.pone.0257243 (PMC8448358; doi:10.1371/journal.pone.0257243)

## Authentic KSK crude drugs

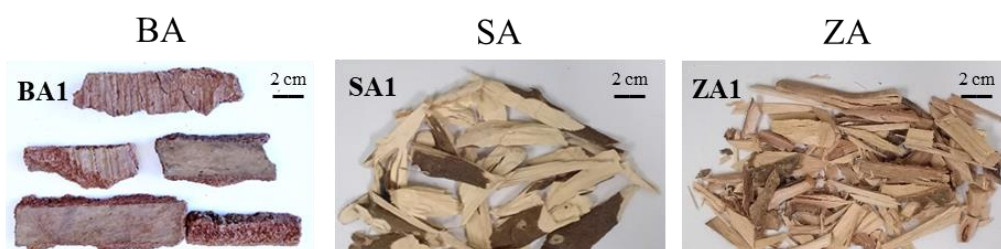

## Commercial KSK crude drugs

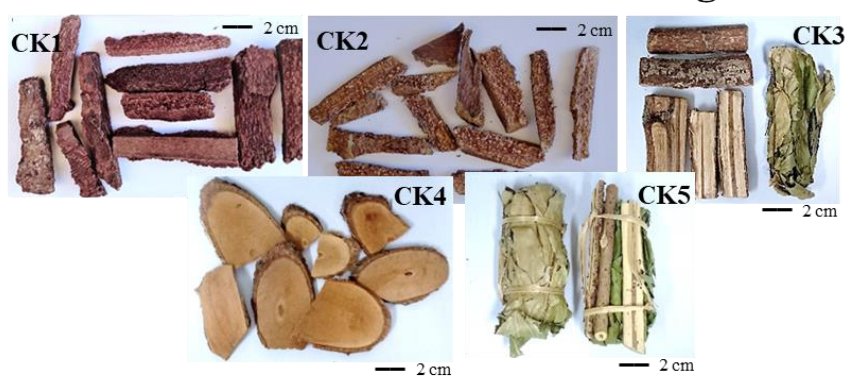

**S2 Fig. KSK crude drugs used in this study.**

Supplement: S2 Fig — (PDF) [file pone.0257243.s002.pdf]

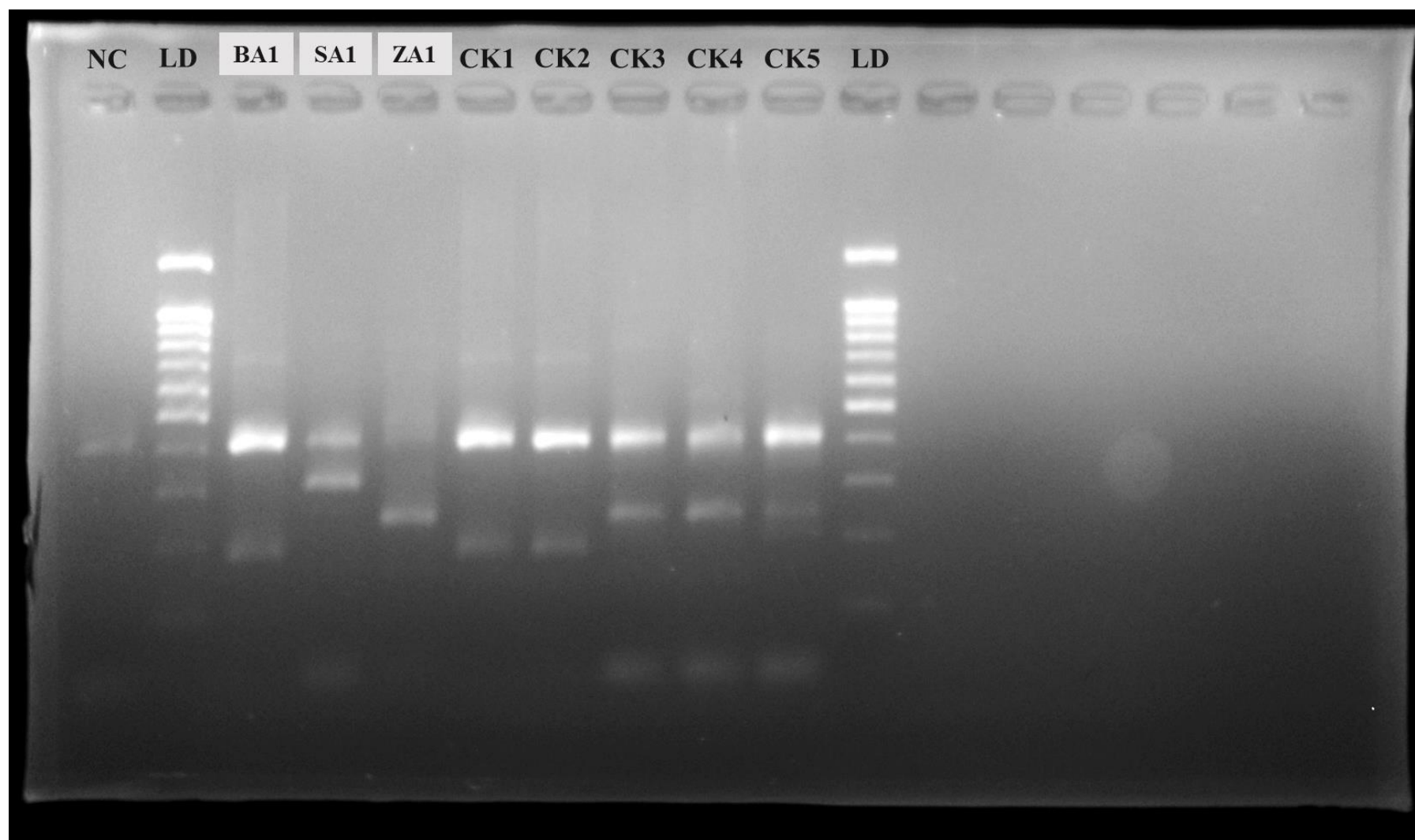

**S3 Fig.** The original image of the amplicons from multiplex PCR represented in Figure 6A.

Supplement: S3 Fig — (PDF) [file pone.0257243.s003.pdf]
