# Supplementary material for: Verification of Thai ethnobotanical medicine “Kamlang Suea Khrong” driven by multiplex PCR and powerful TLC techniques
Source: PLoS One. 2021 Sep 17;16(9):e0257243. doi: 10.1371/journal.pone.0257243 (PMC8448358; doi:10.1371/journal.pone.0257243)
Supplement: S1 Appendix — (PDF) [file pone.0257243.s004.pdf]

S1 Appendix. The authentic KSK plants collected in this study.

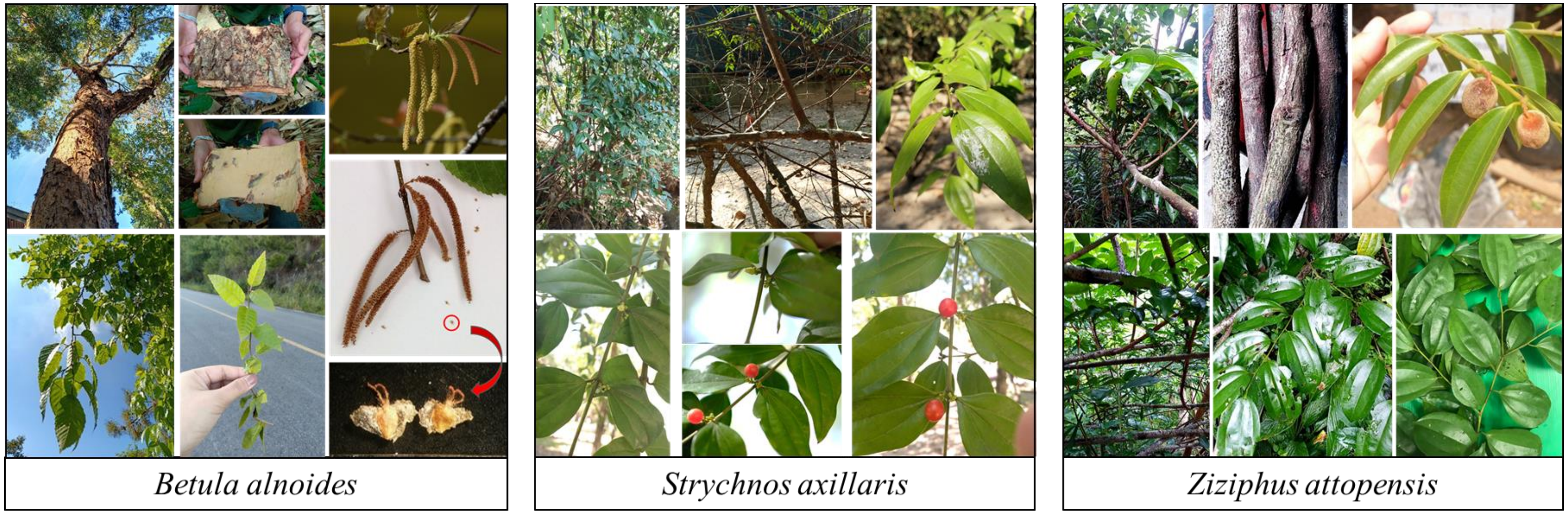

S1 Fig A. The authentic plants used in this study.

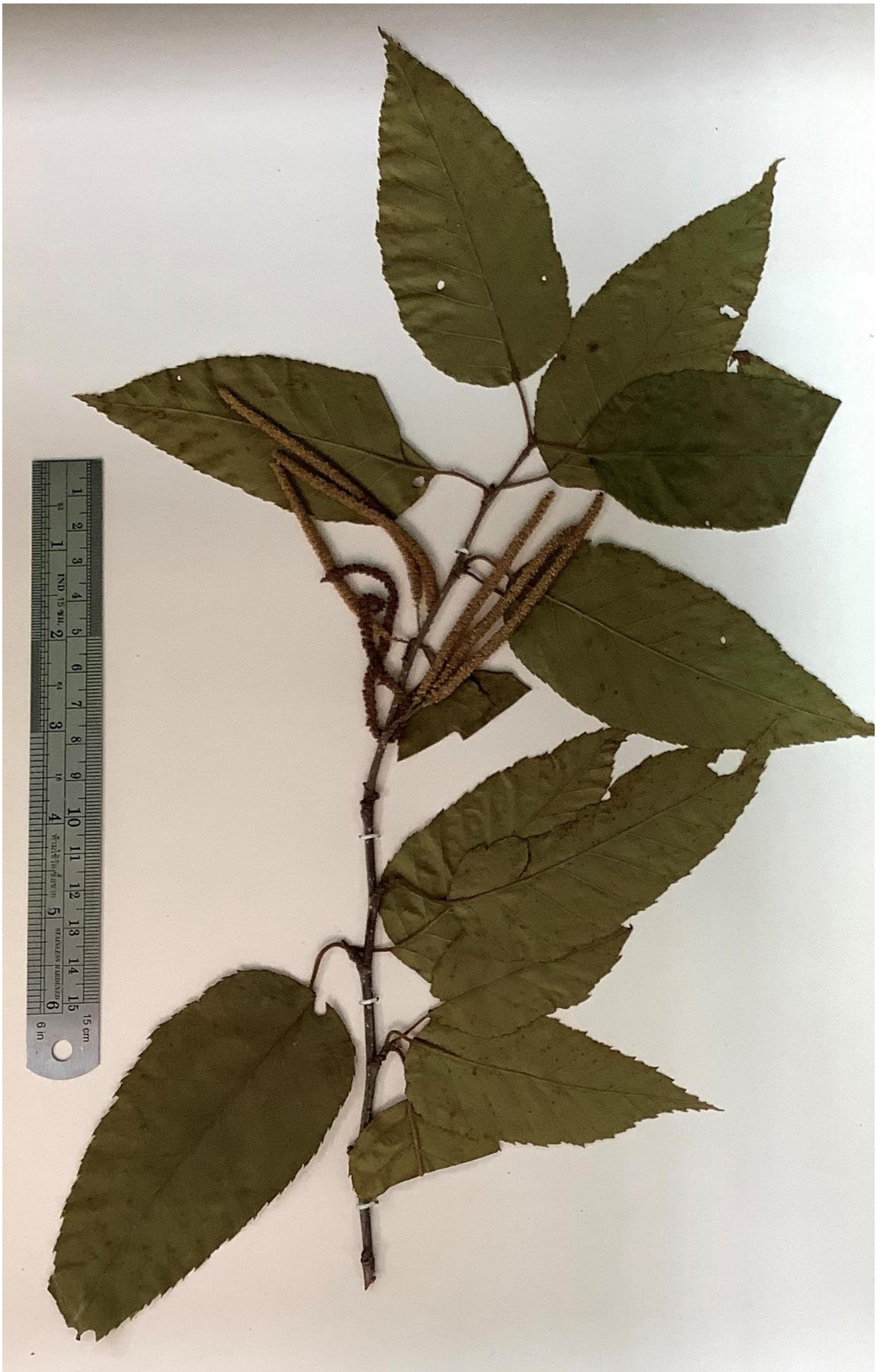

S1 Fig B. BA1 specimen.

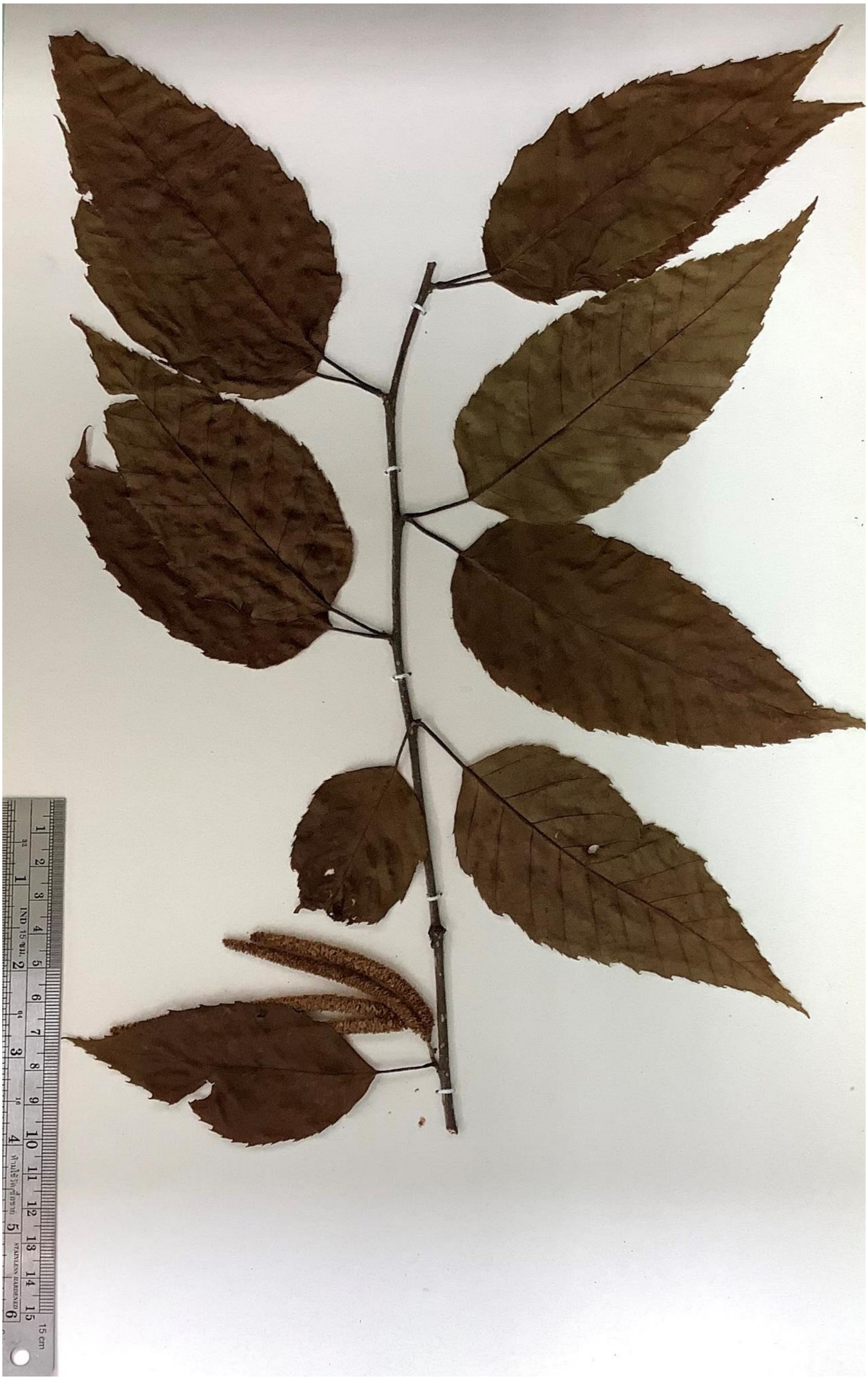

S1 Fig C. BA2 specimen.

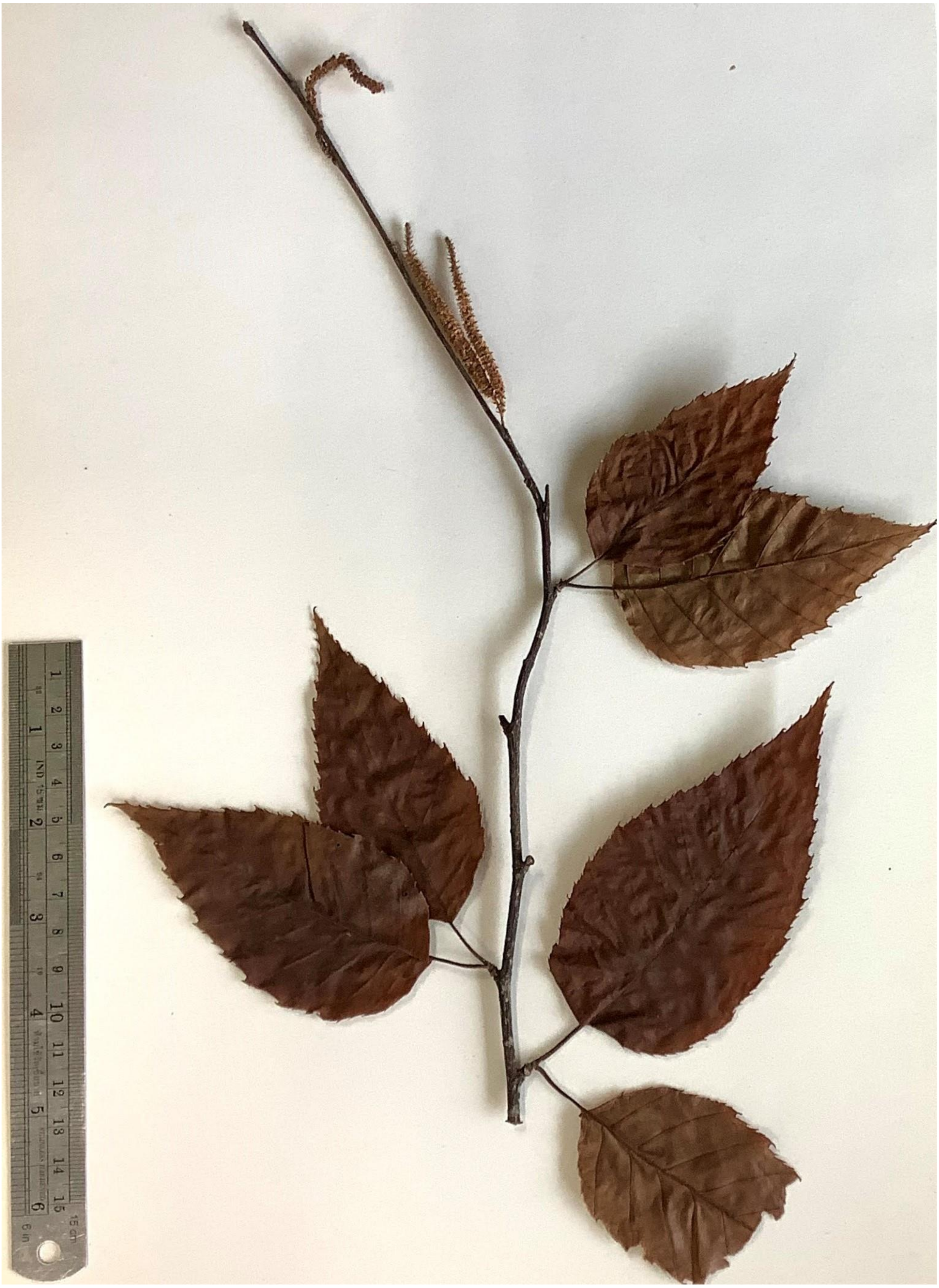

S1 Fig D. BA3 specimen.

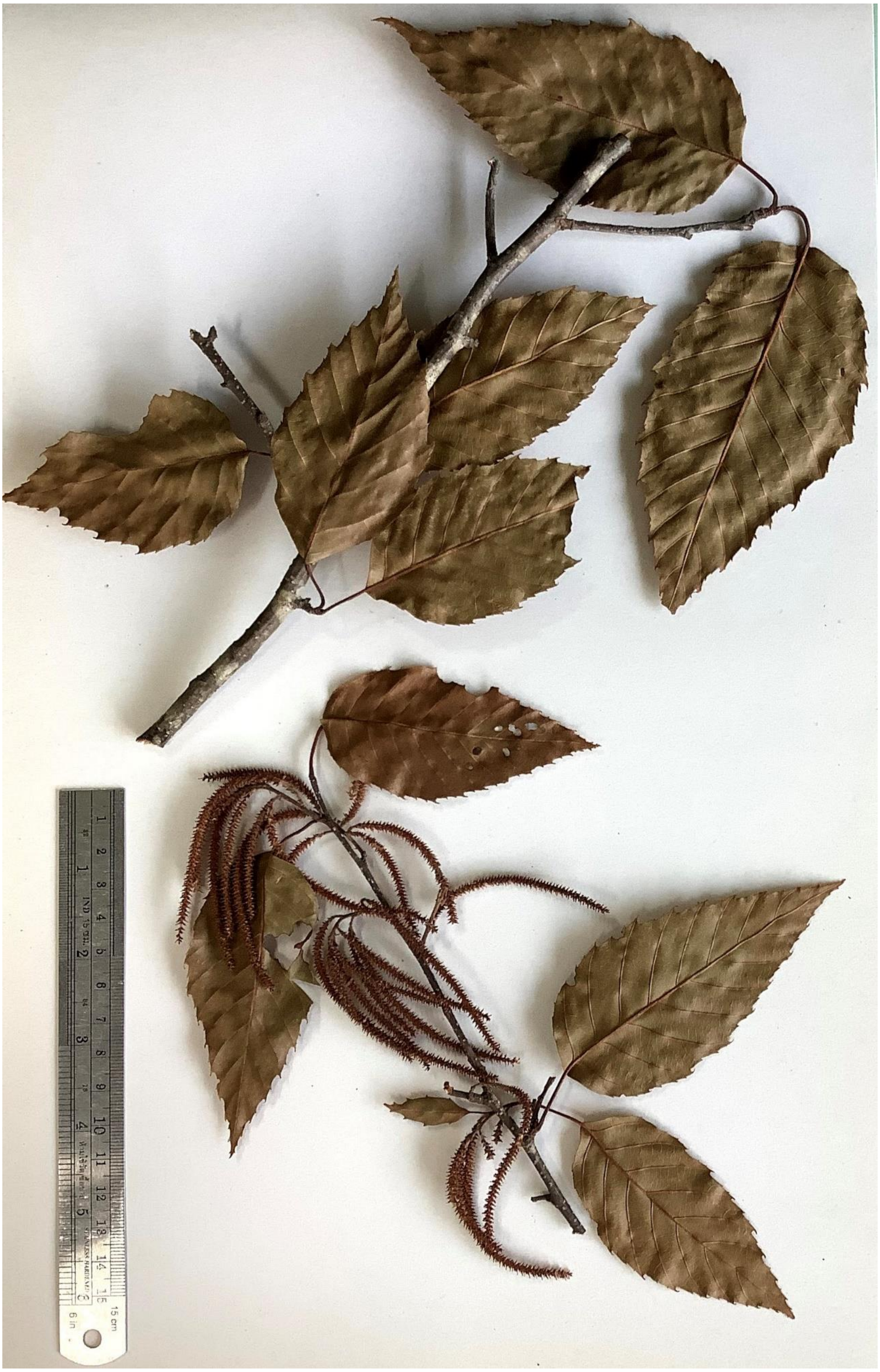

S1 Fig E. BA4 specimen.

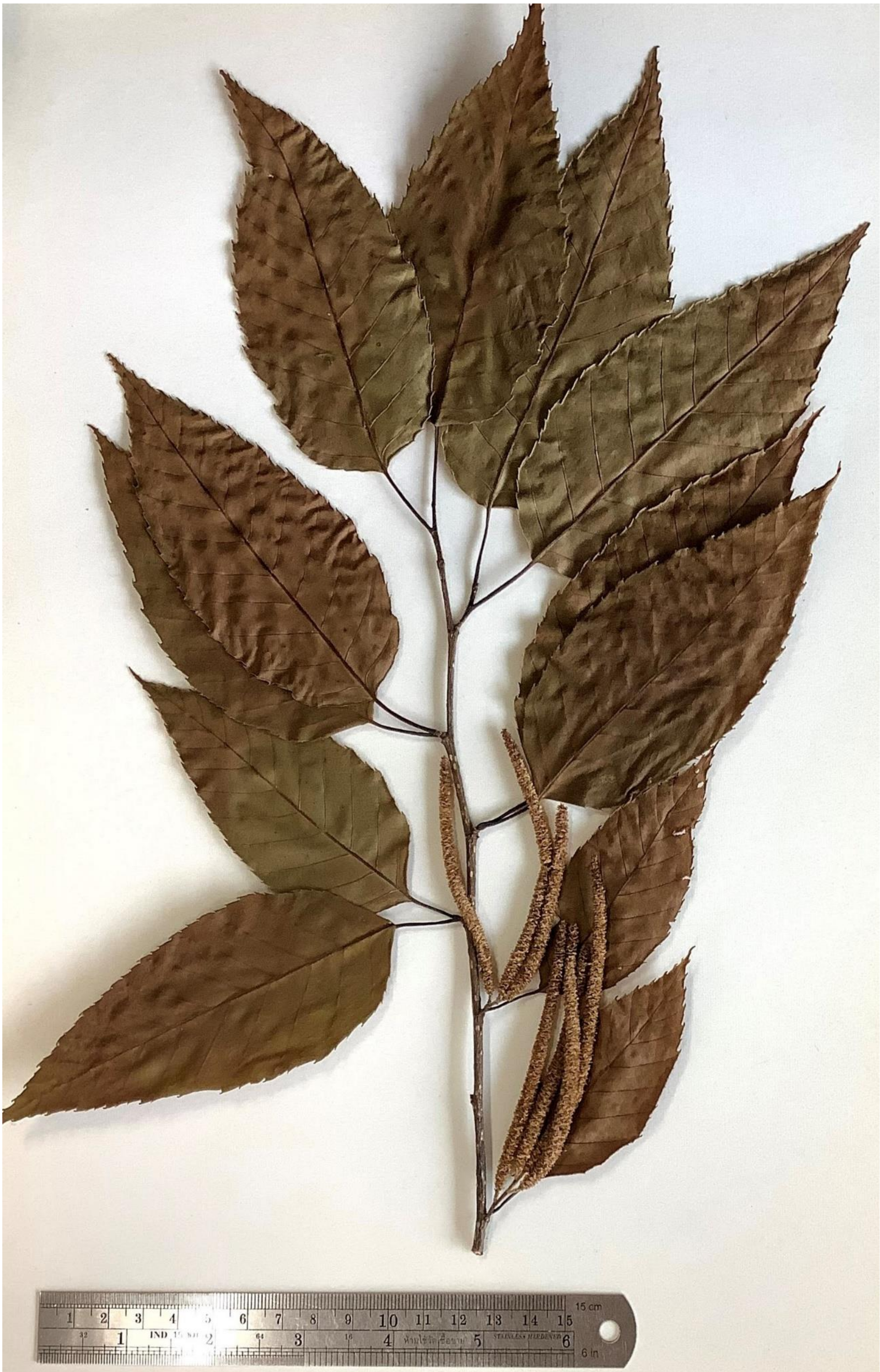

S1 Fig F. BA5 specimen.

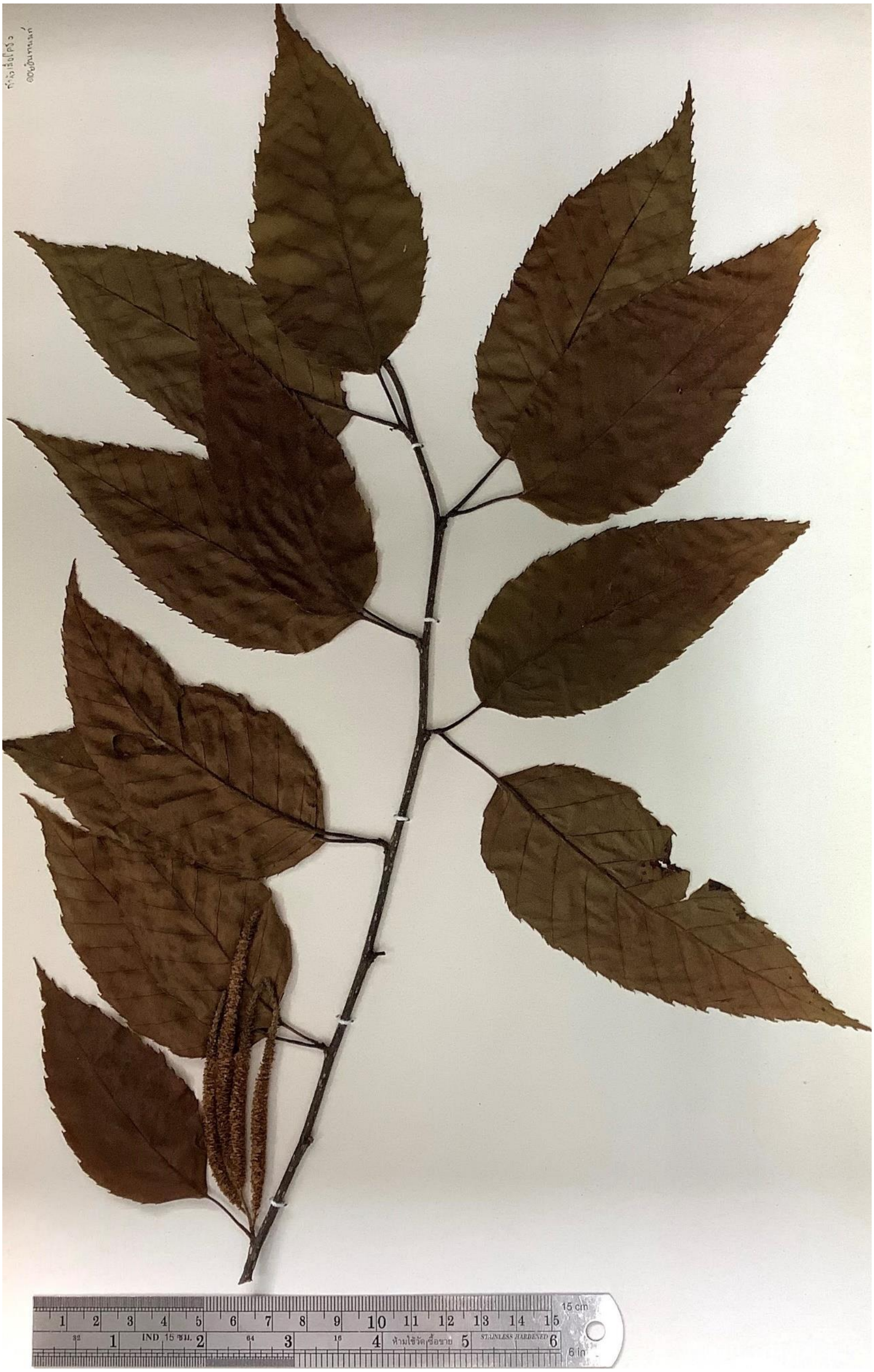

S1 Fig G. BA6 specimen.

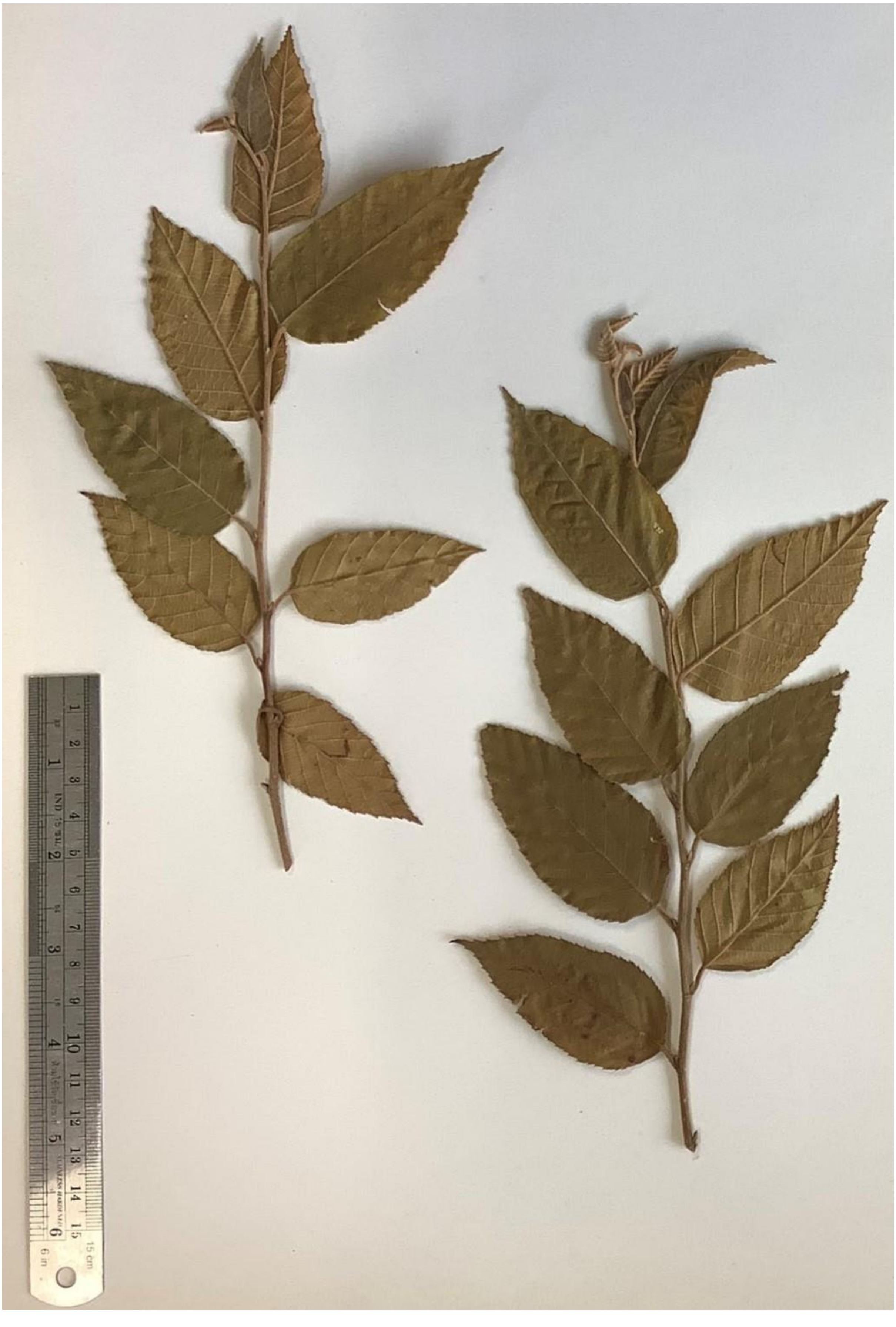

S1 Fig H. BA7 specimen.

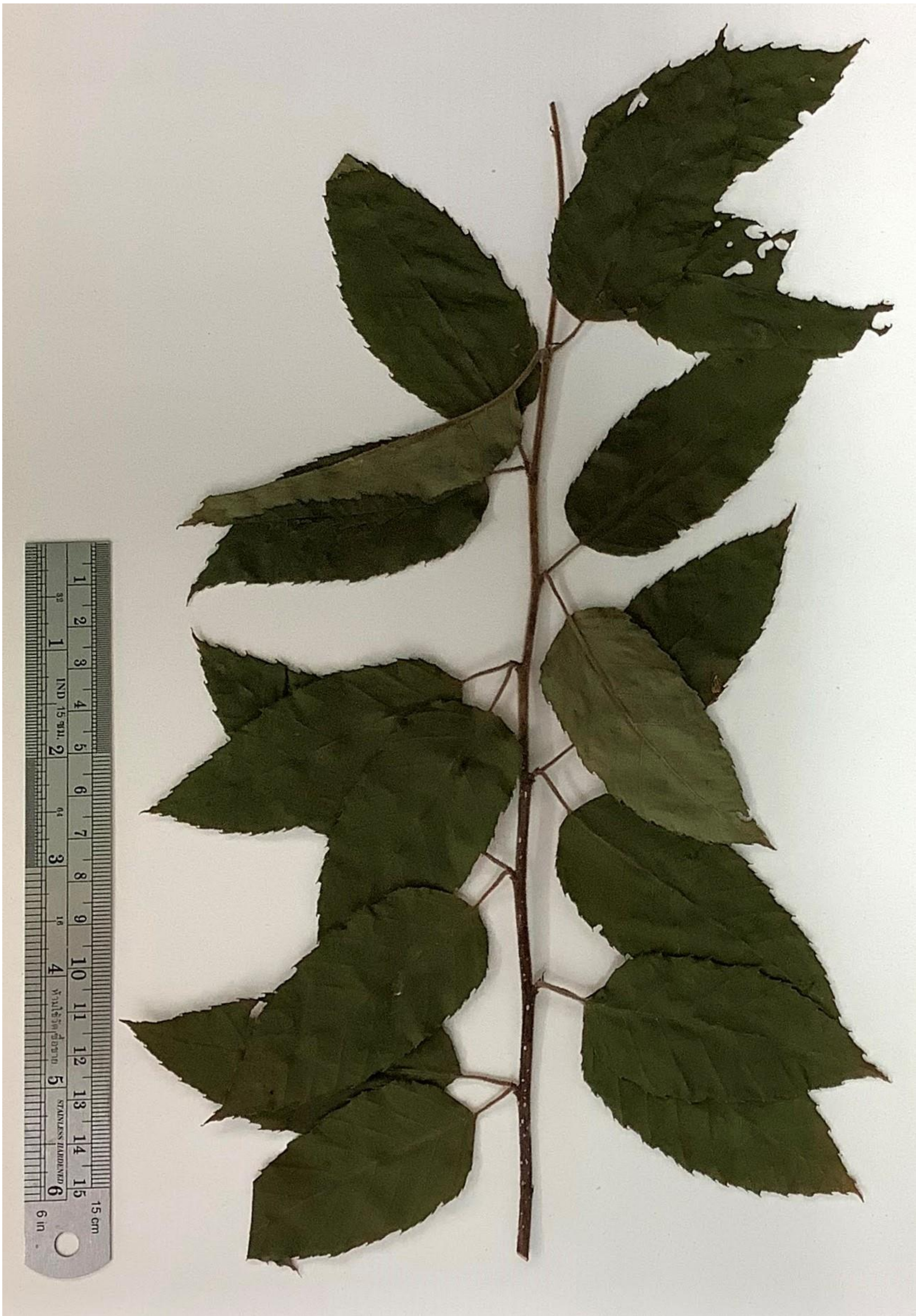

S1 Fig I. BA8 specimen.

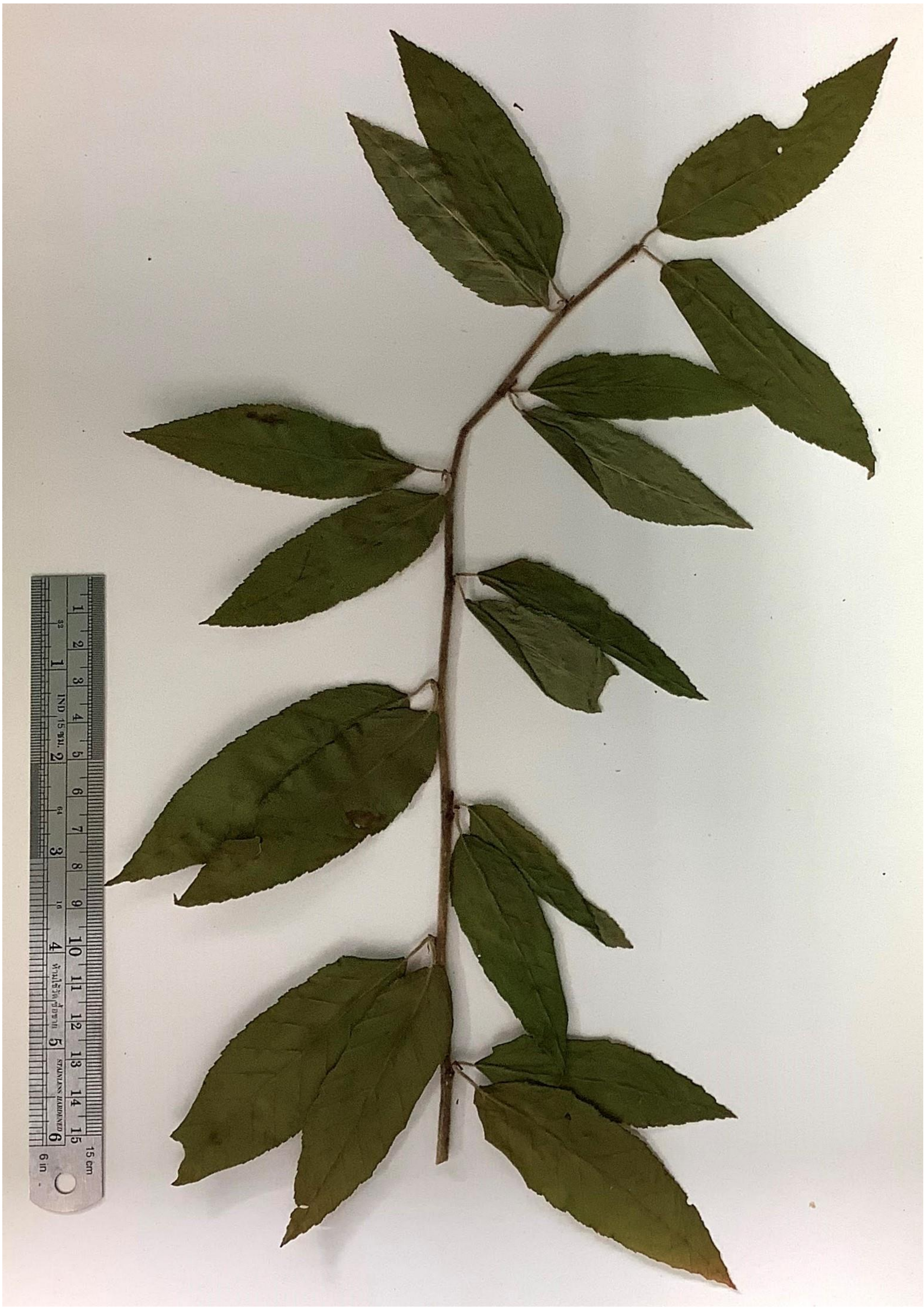

S1 Fig J. BA9 specimen.

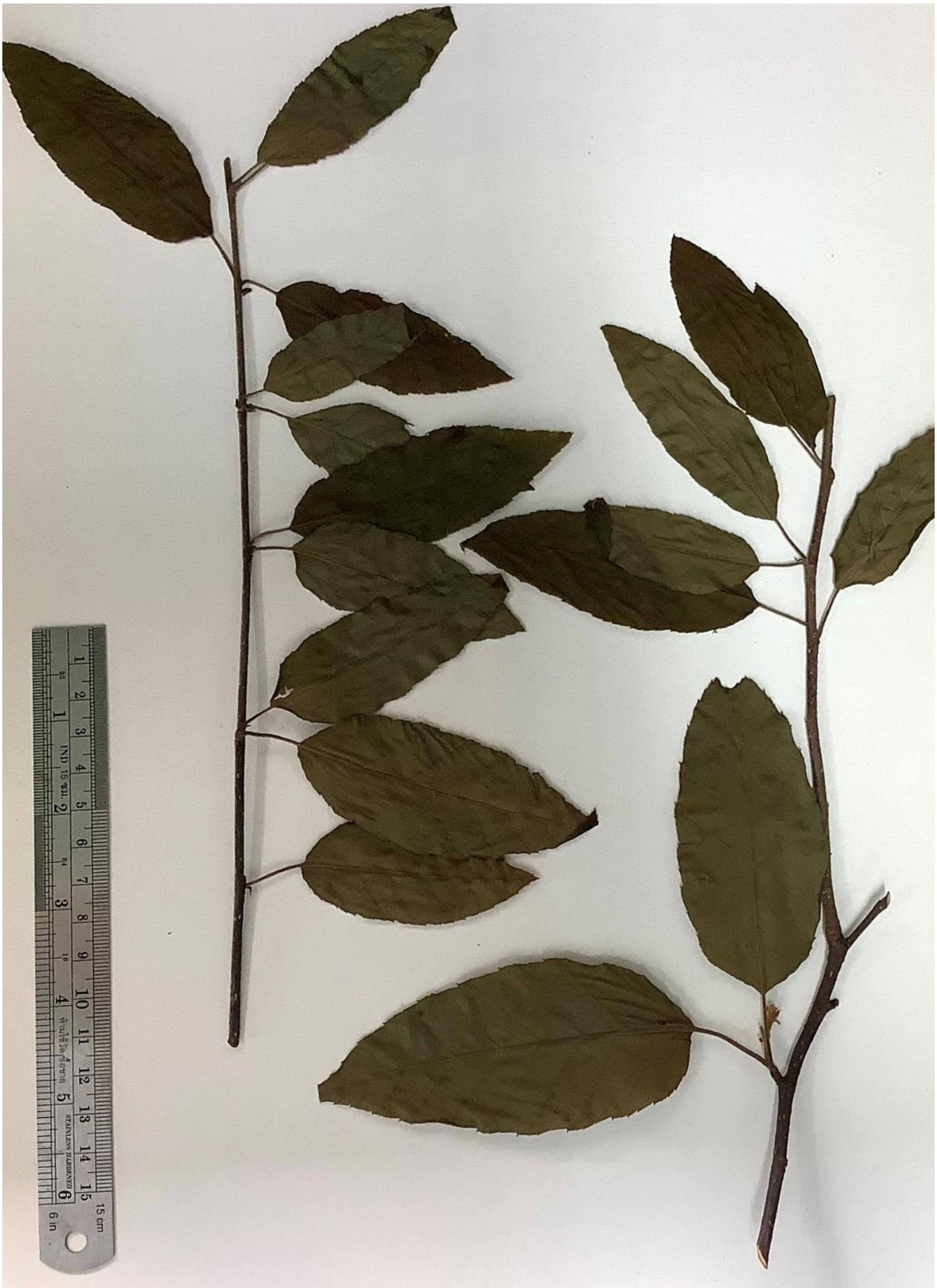

S1 Fig K. BA10 specimen.

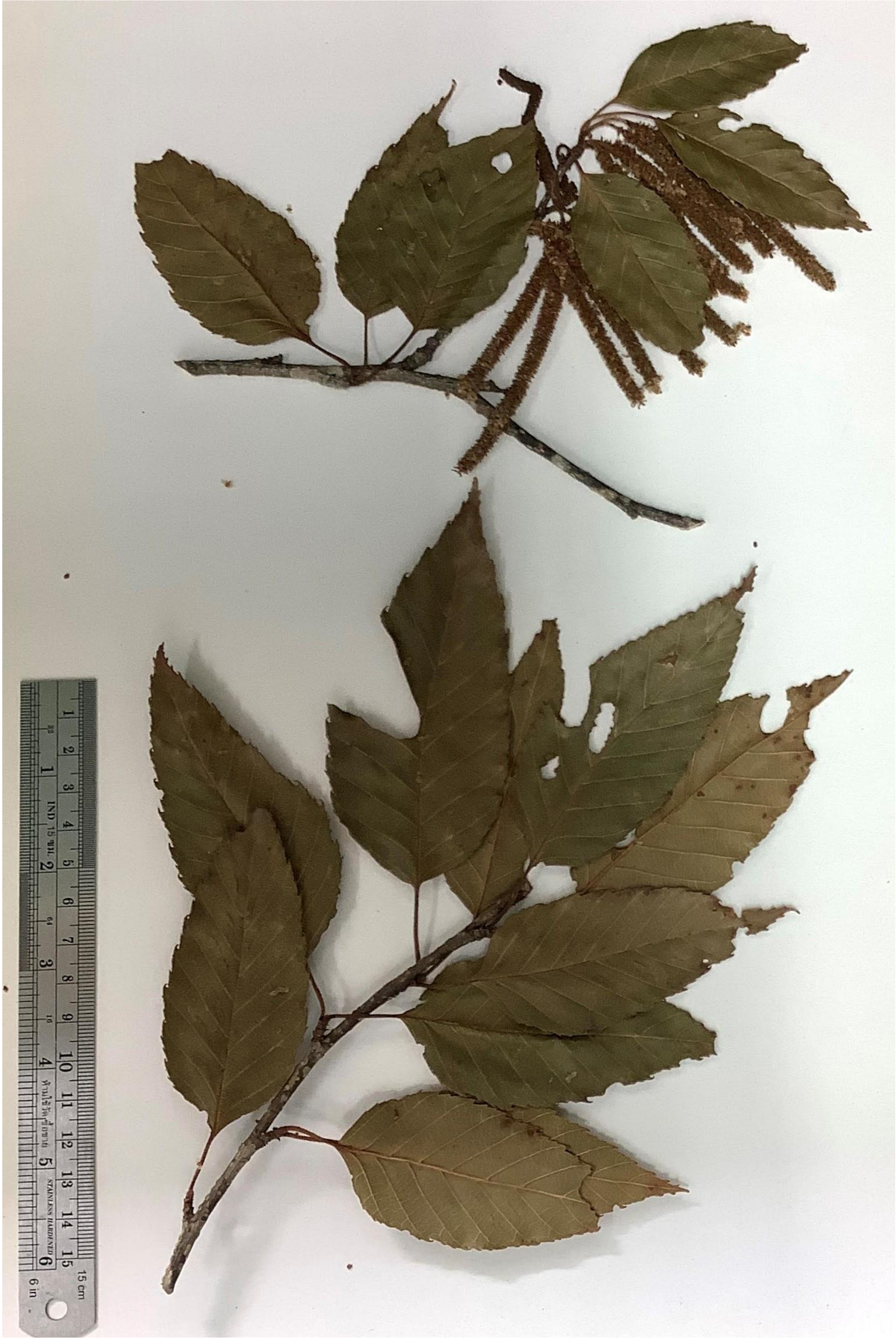

S1 Fig L. BA11 specimen.

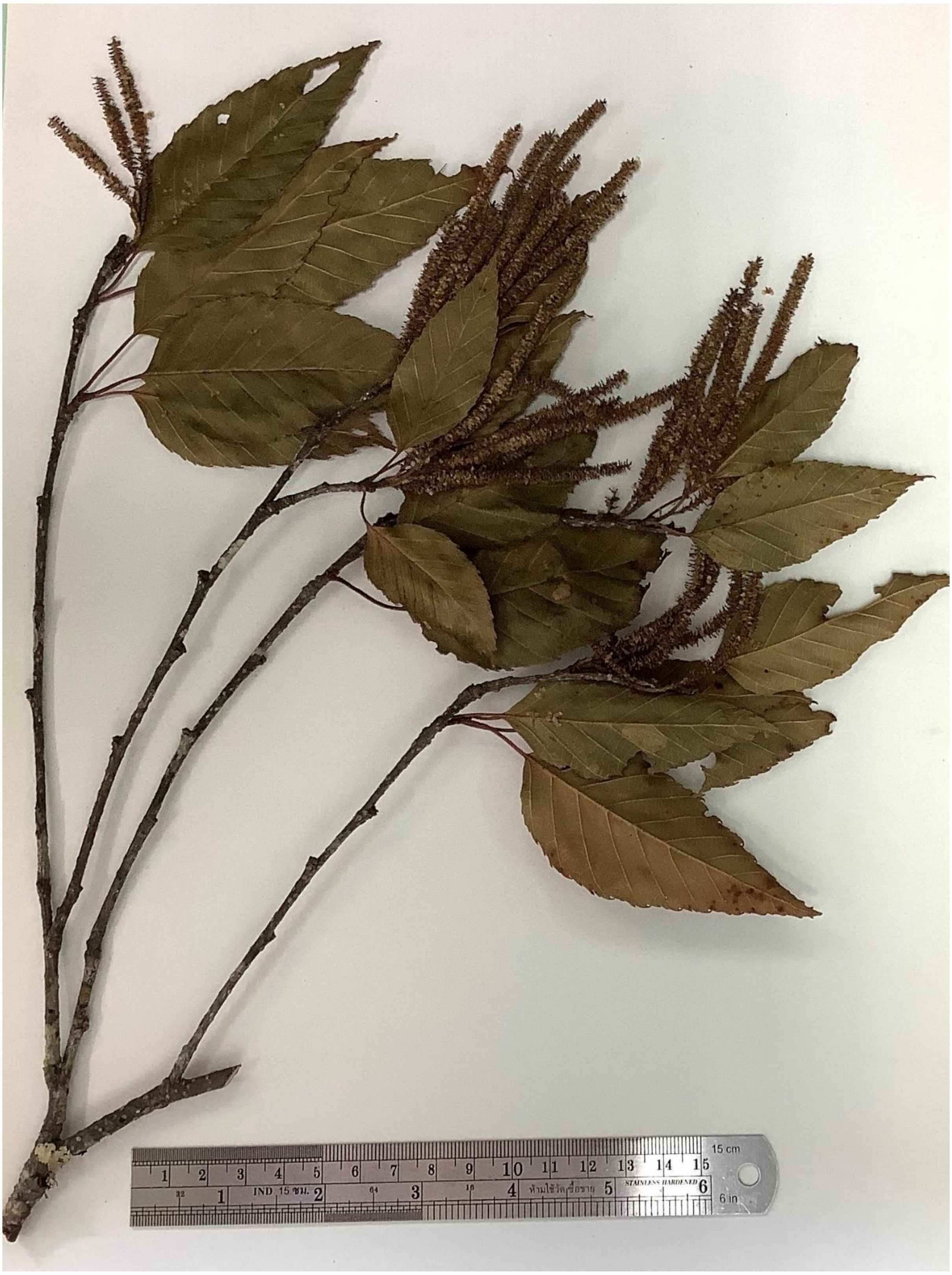

S1 Fig M. BA12 specimen.

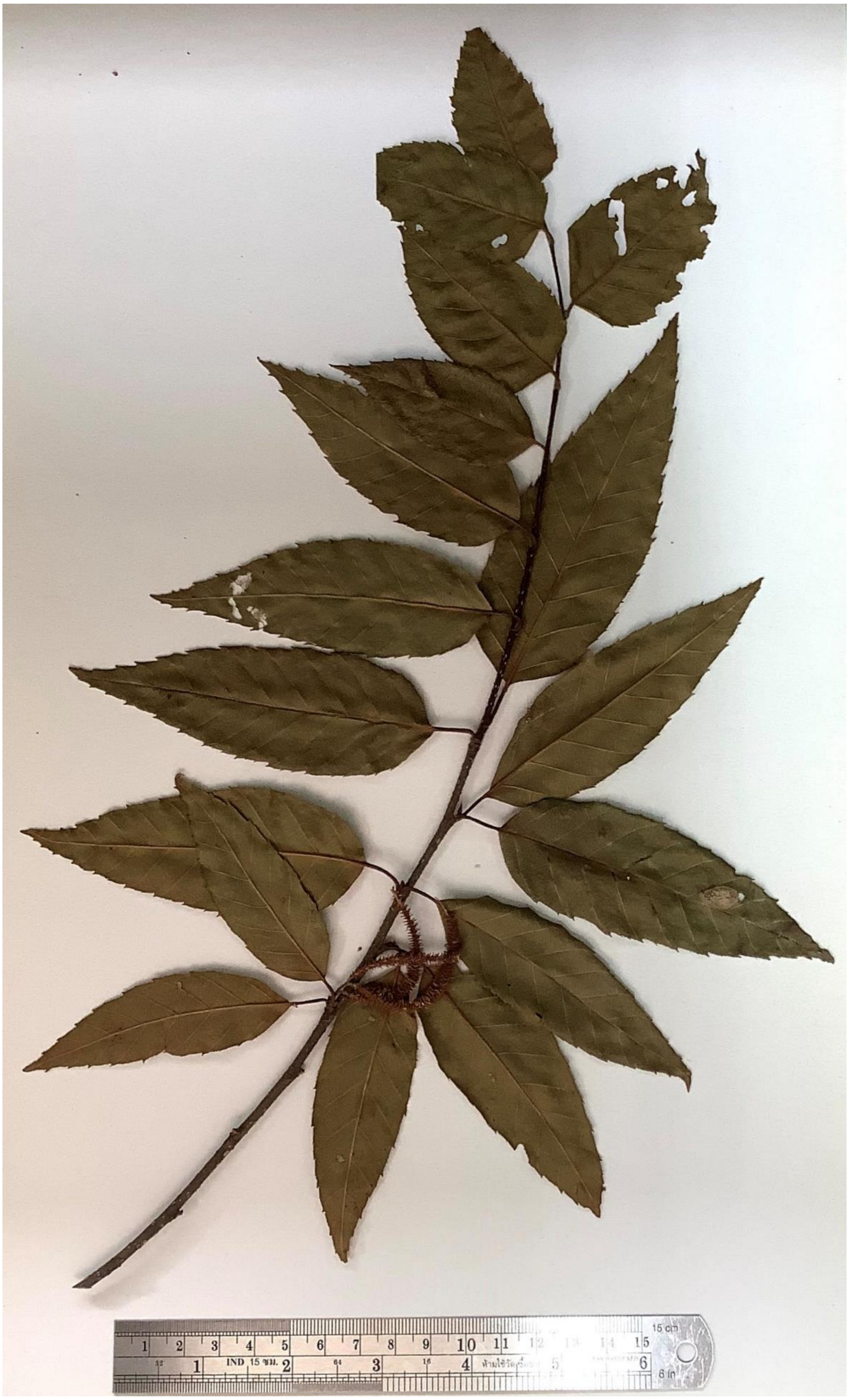

S1 Fig N. BA13 specimen.

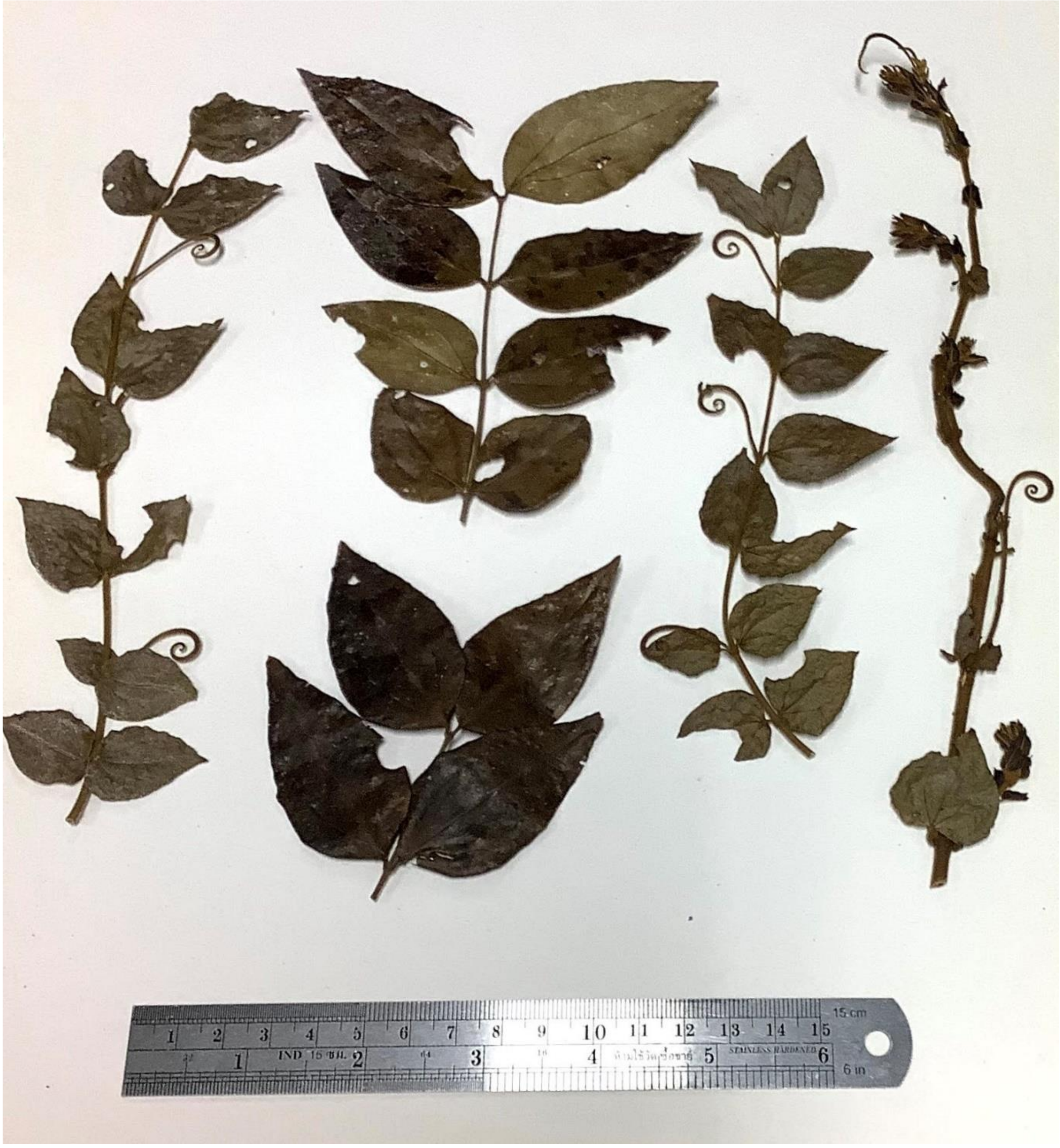

S1 Fig O. SA1 specimen.

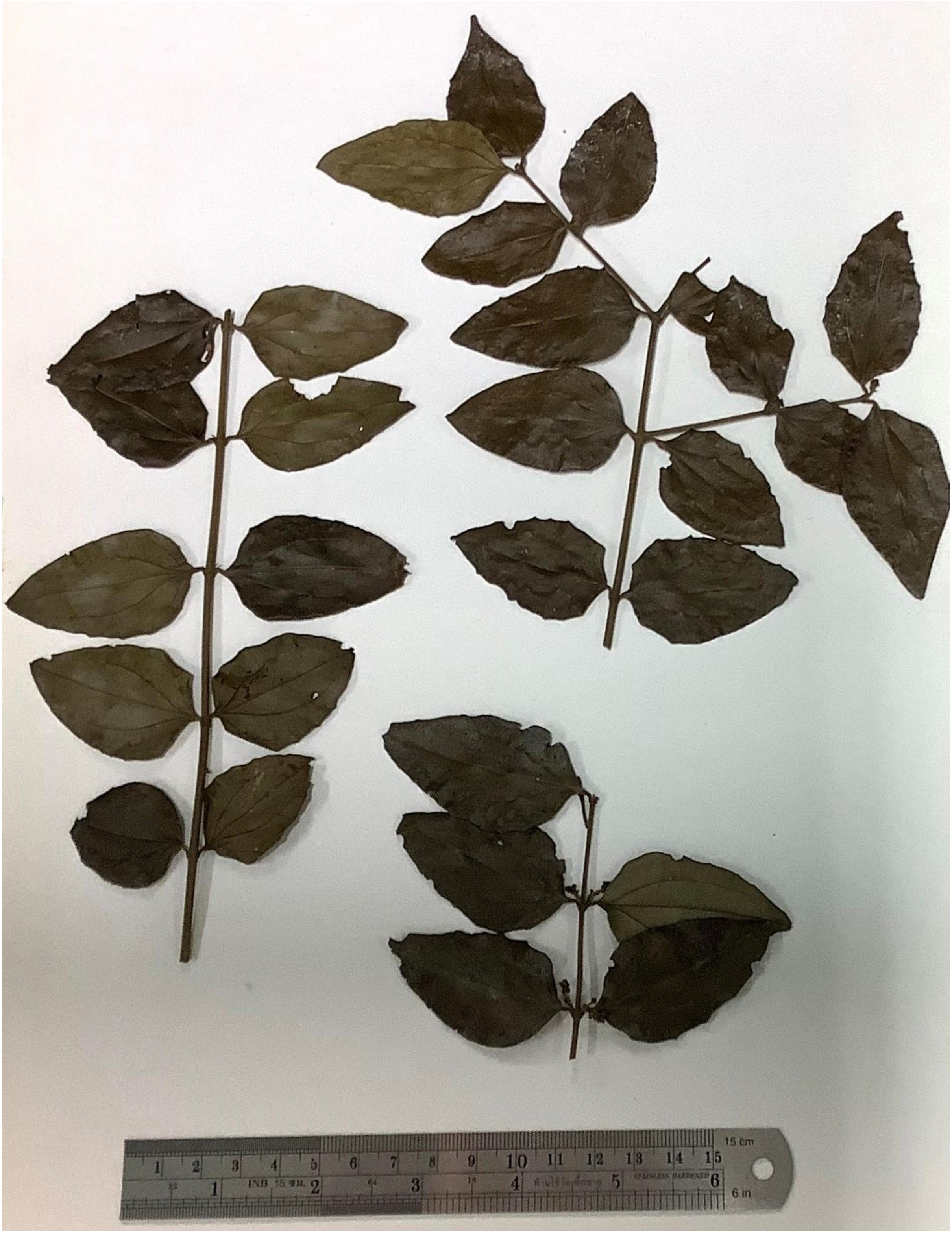

S1 Fig P. SA2 specimen.

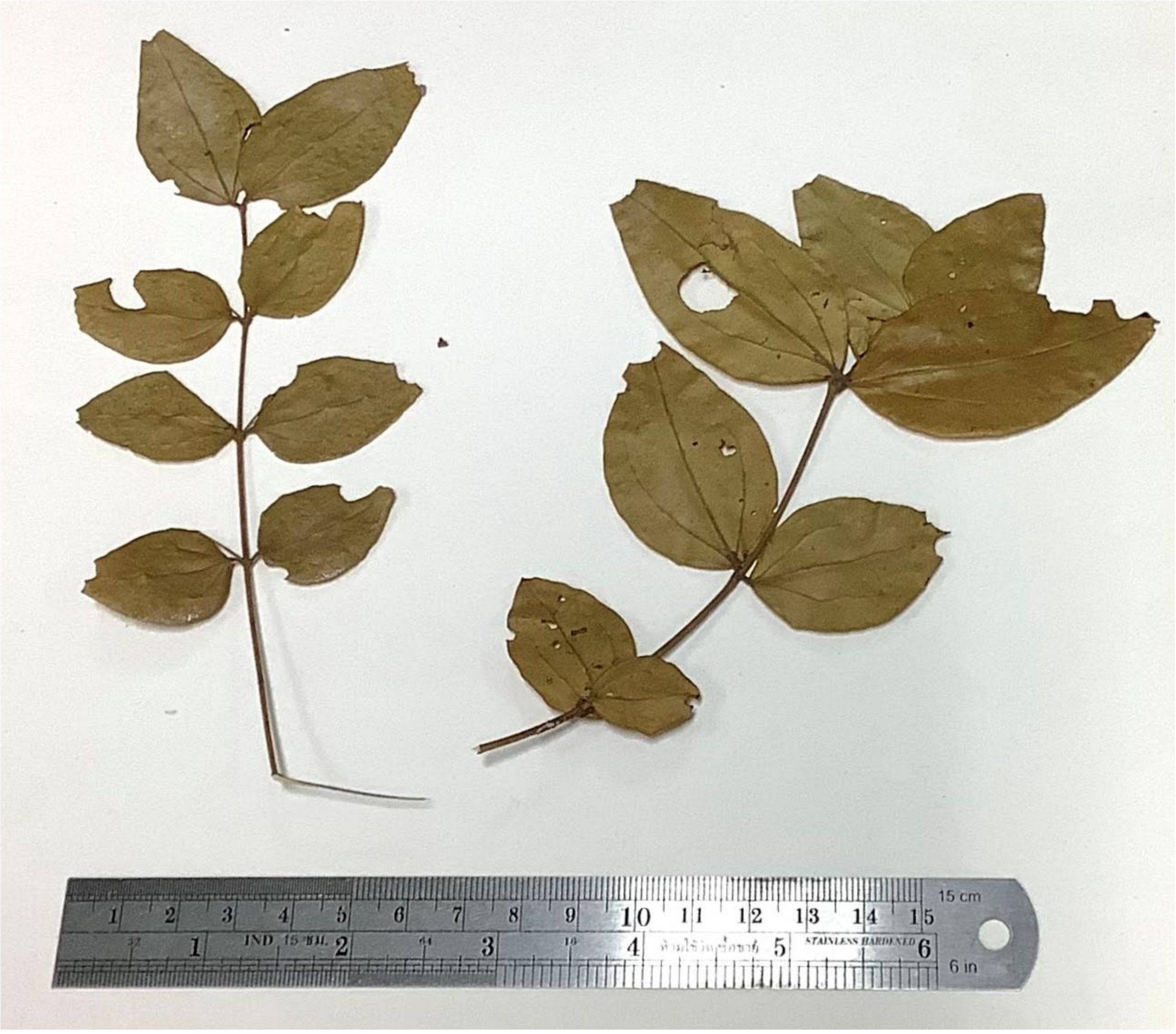

S1 Fig Q. SA3 specimen.

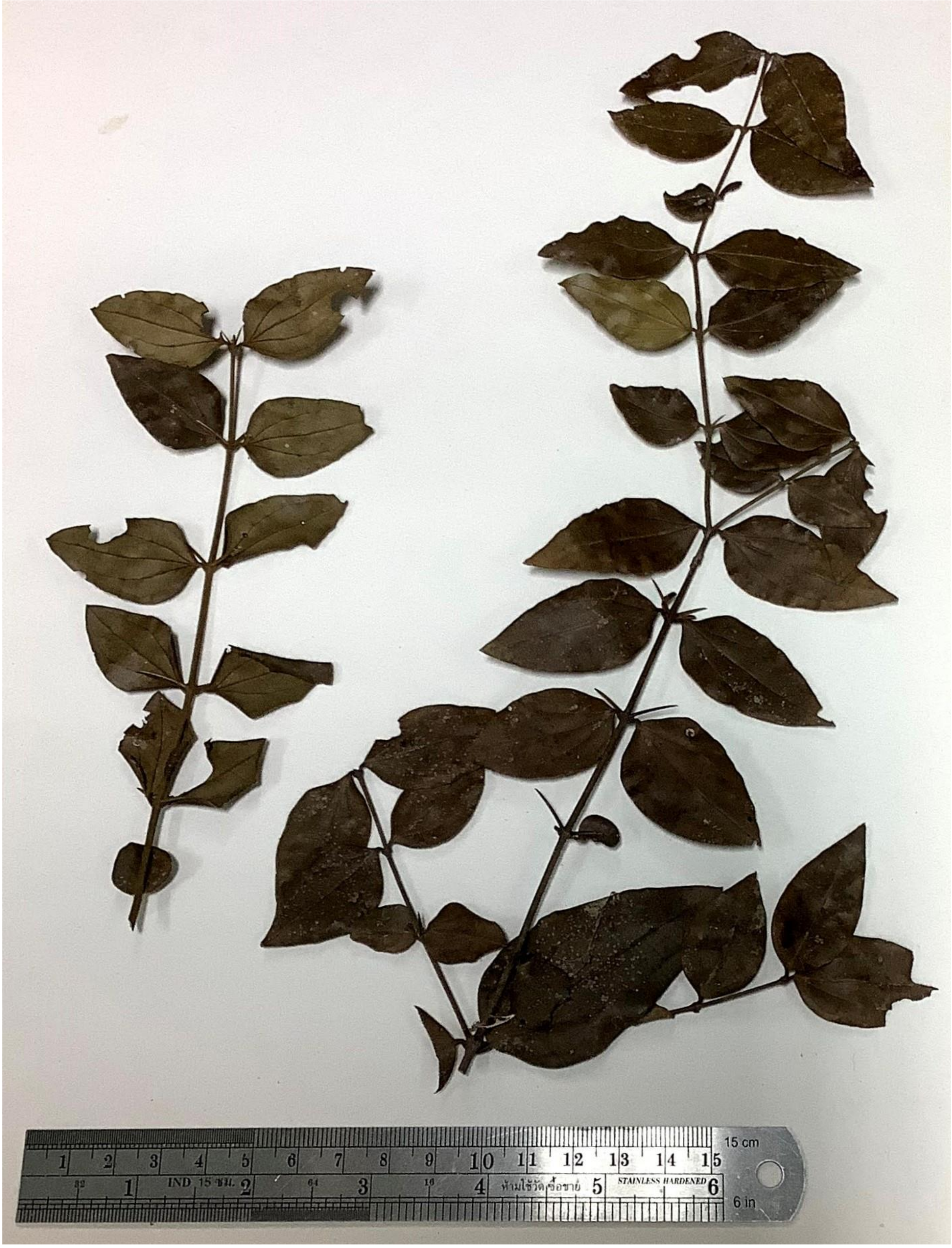

S1 Fig R. SA4 specimen.

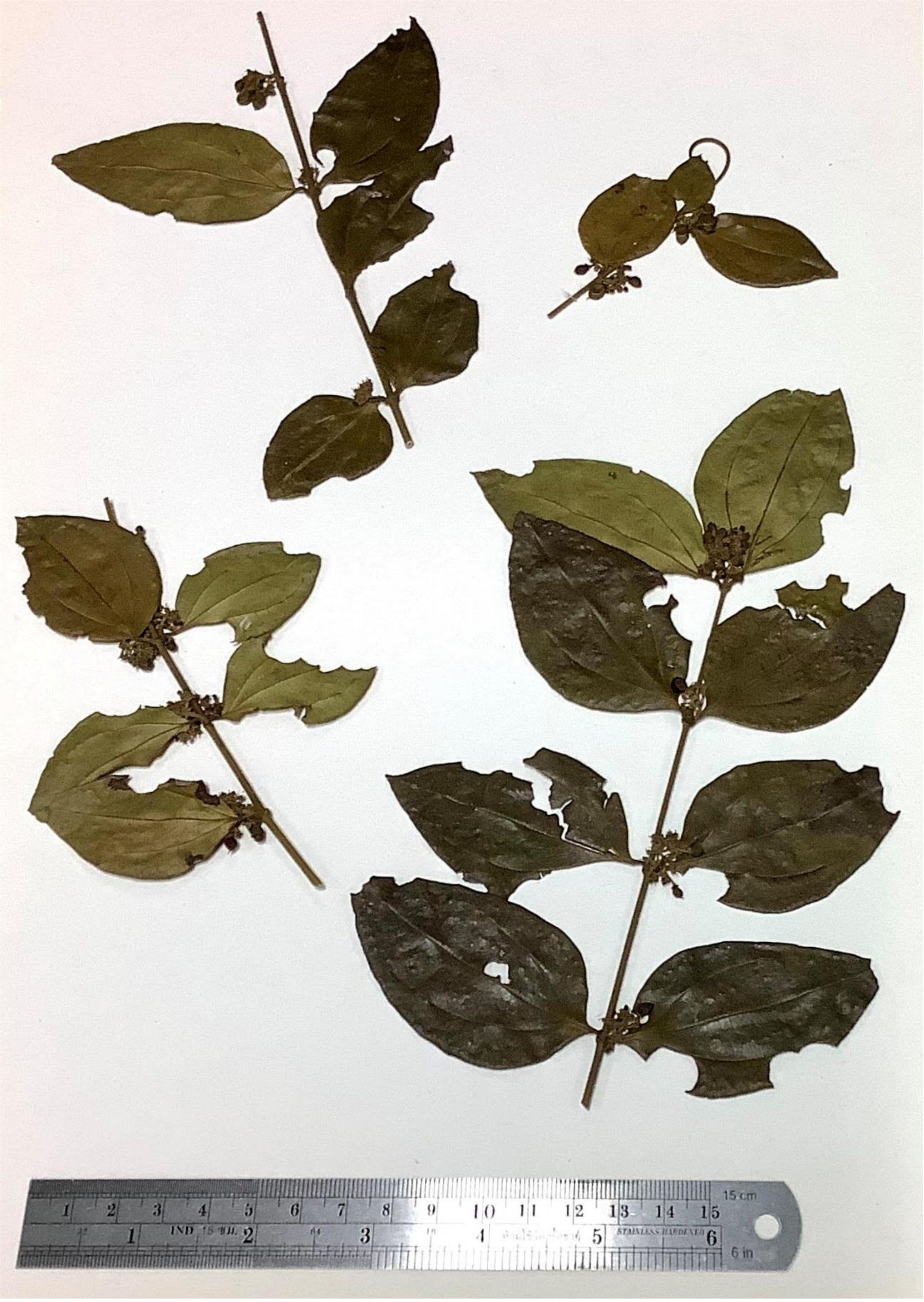

S1 Fig S. SA5 specimen.

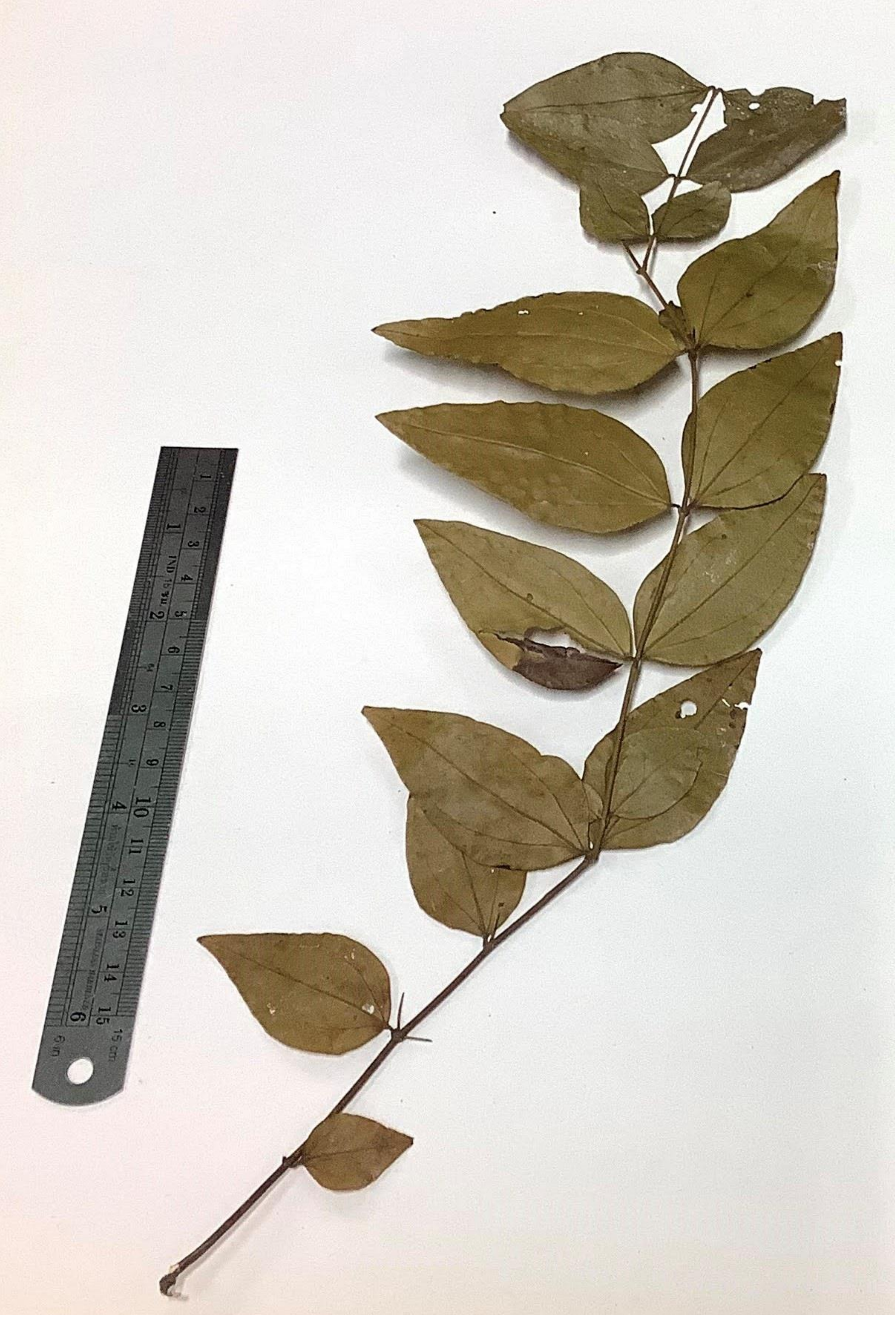

S1 Fig T. SA6 specimen.

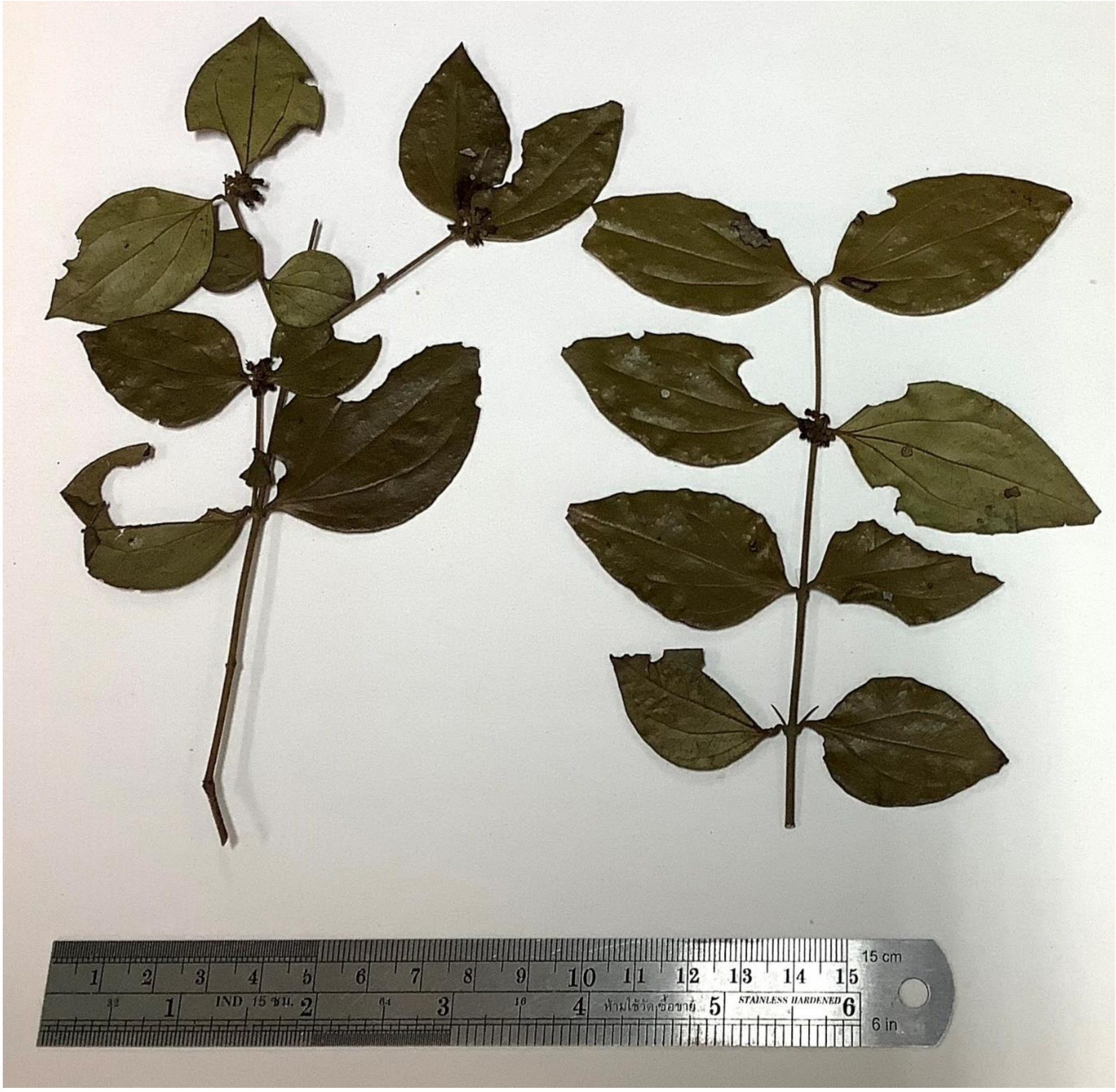

S1 Fig U. SA7 specimen.

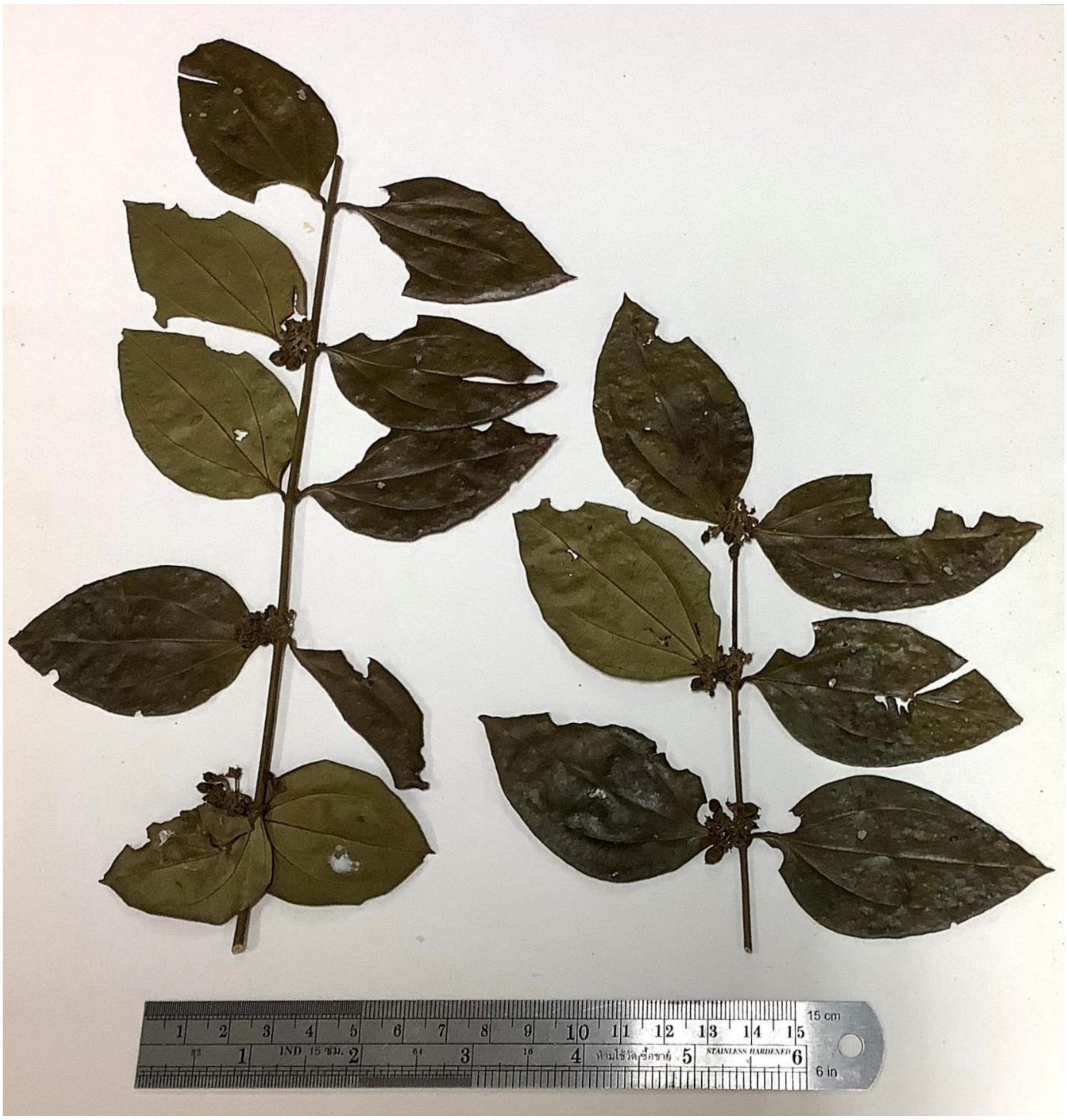

S1 Fig V. SA8 specimen.

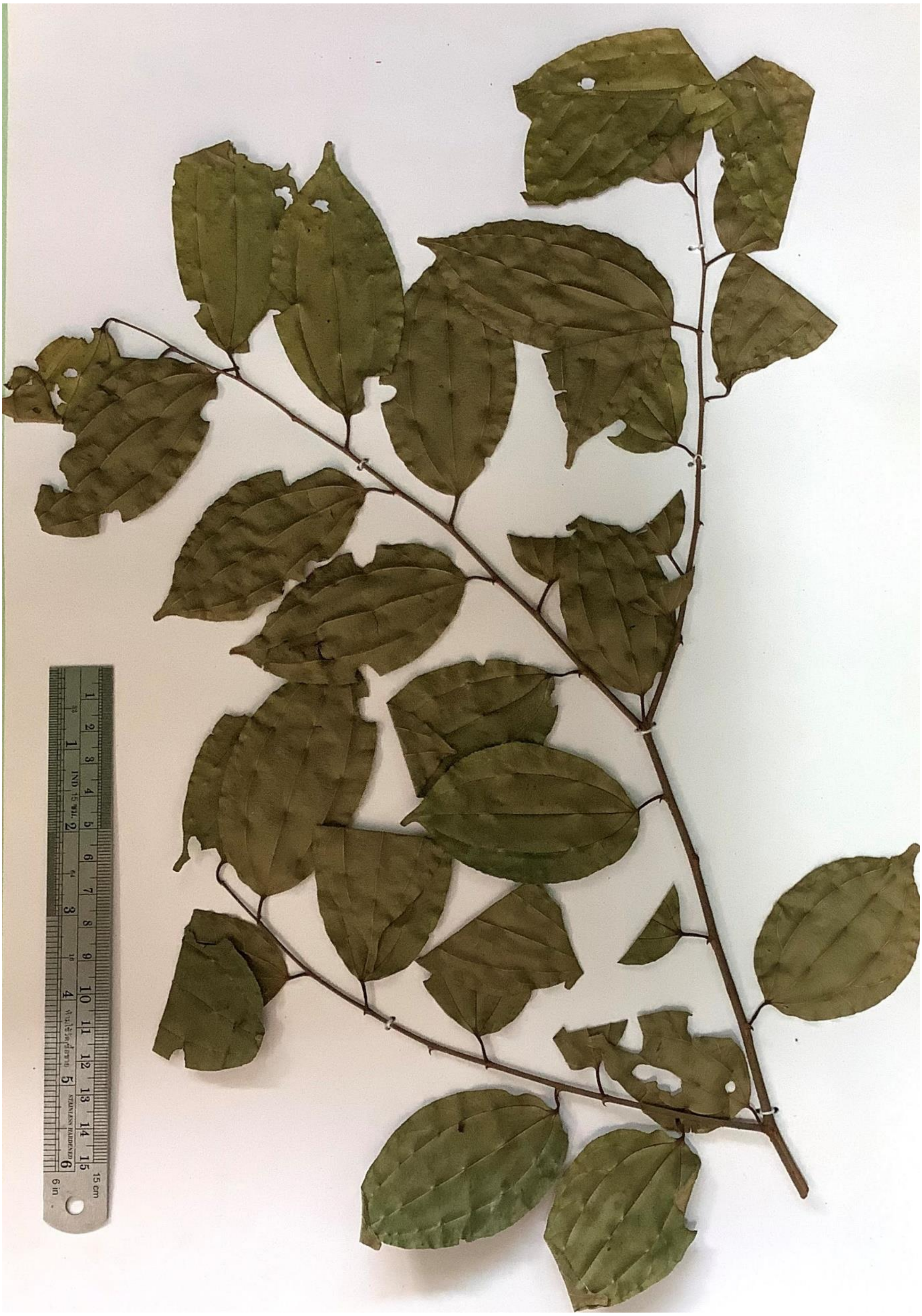

S1 Fig W. ZA1 specimen.

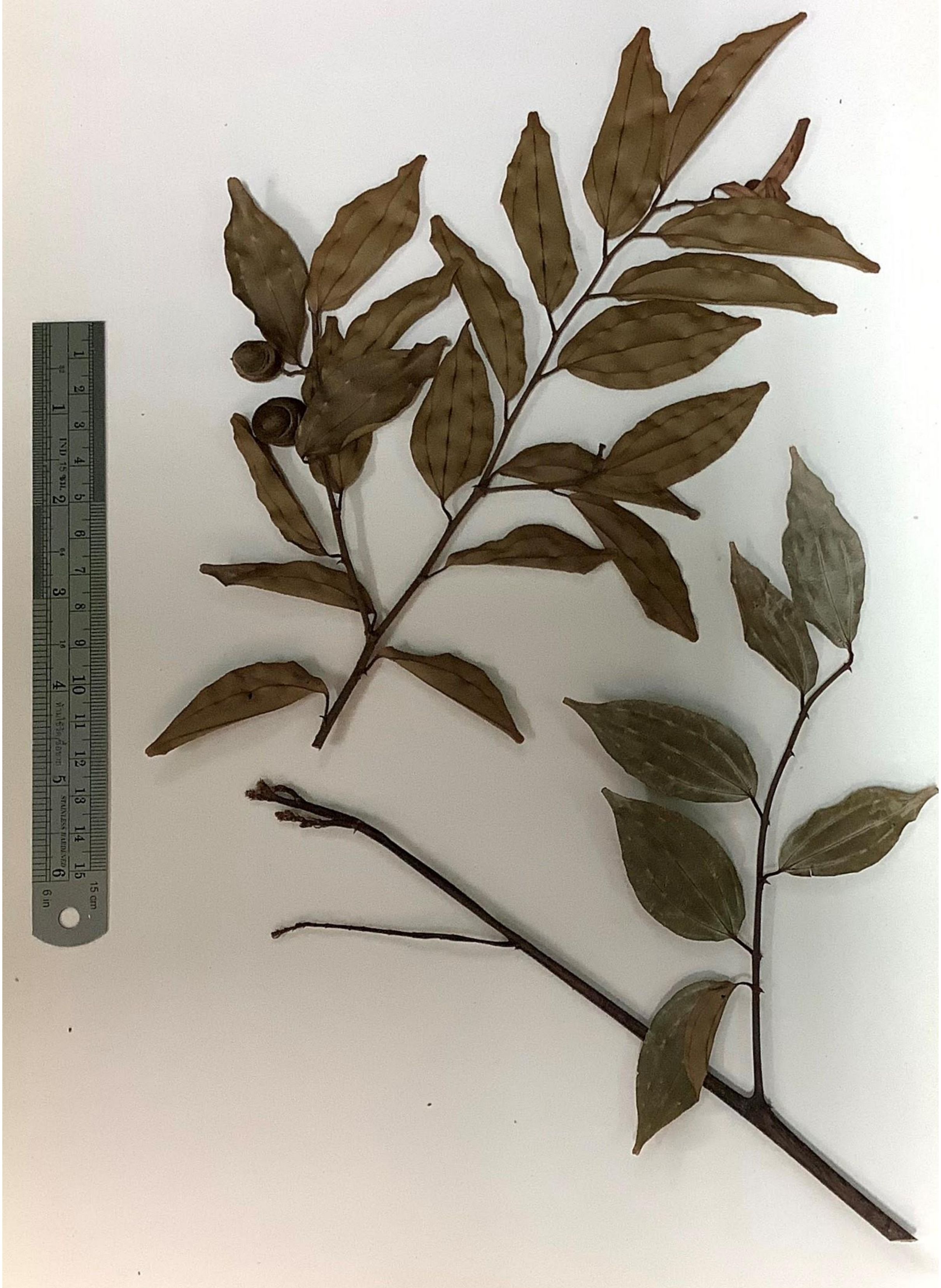

S1 Fig X. ZA2 specimen.

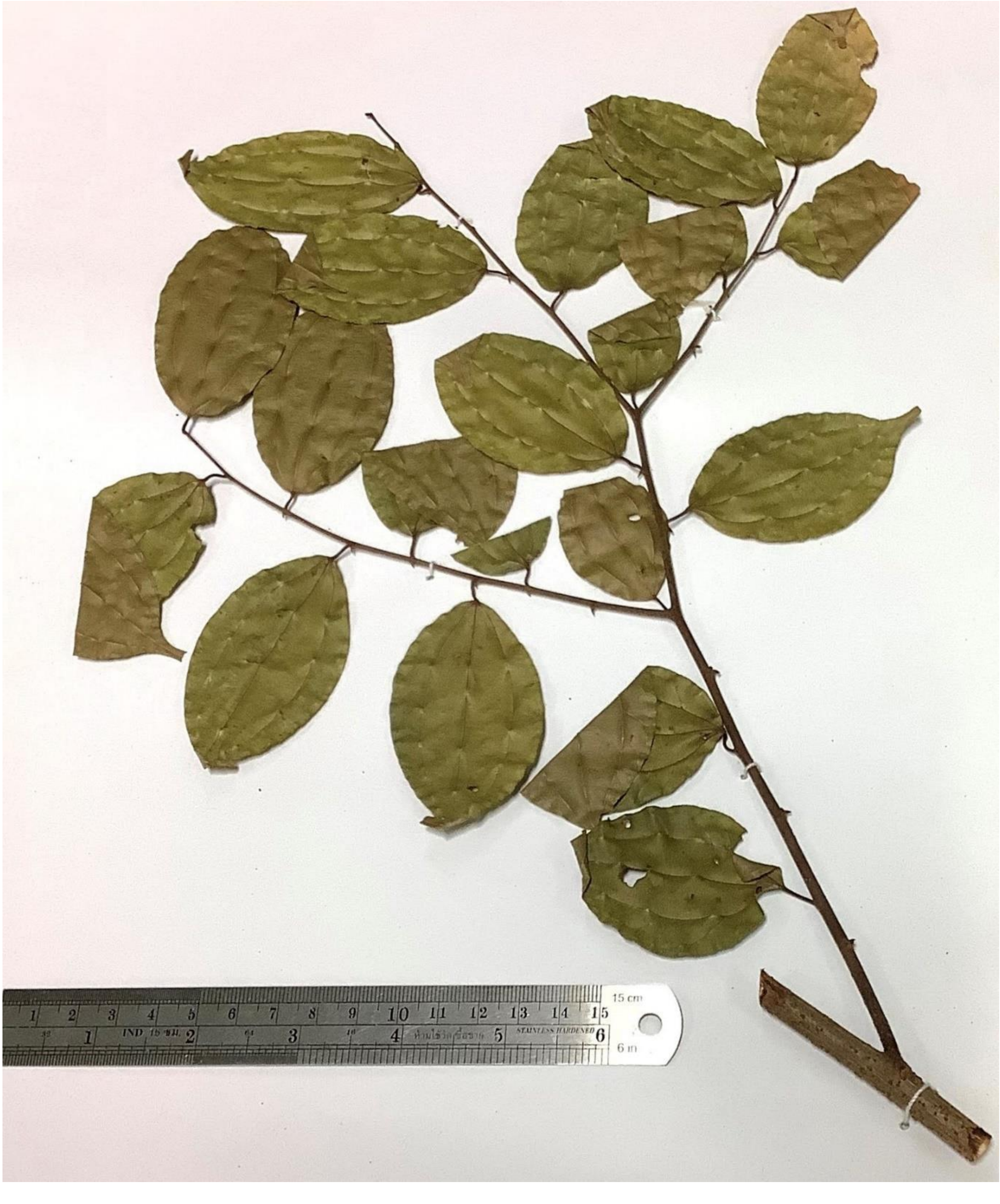

S1 Fig Y. ZA3 specimen.

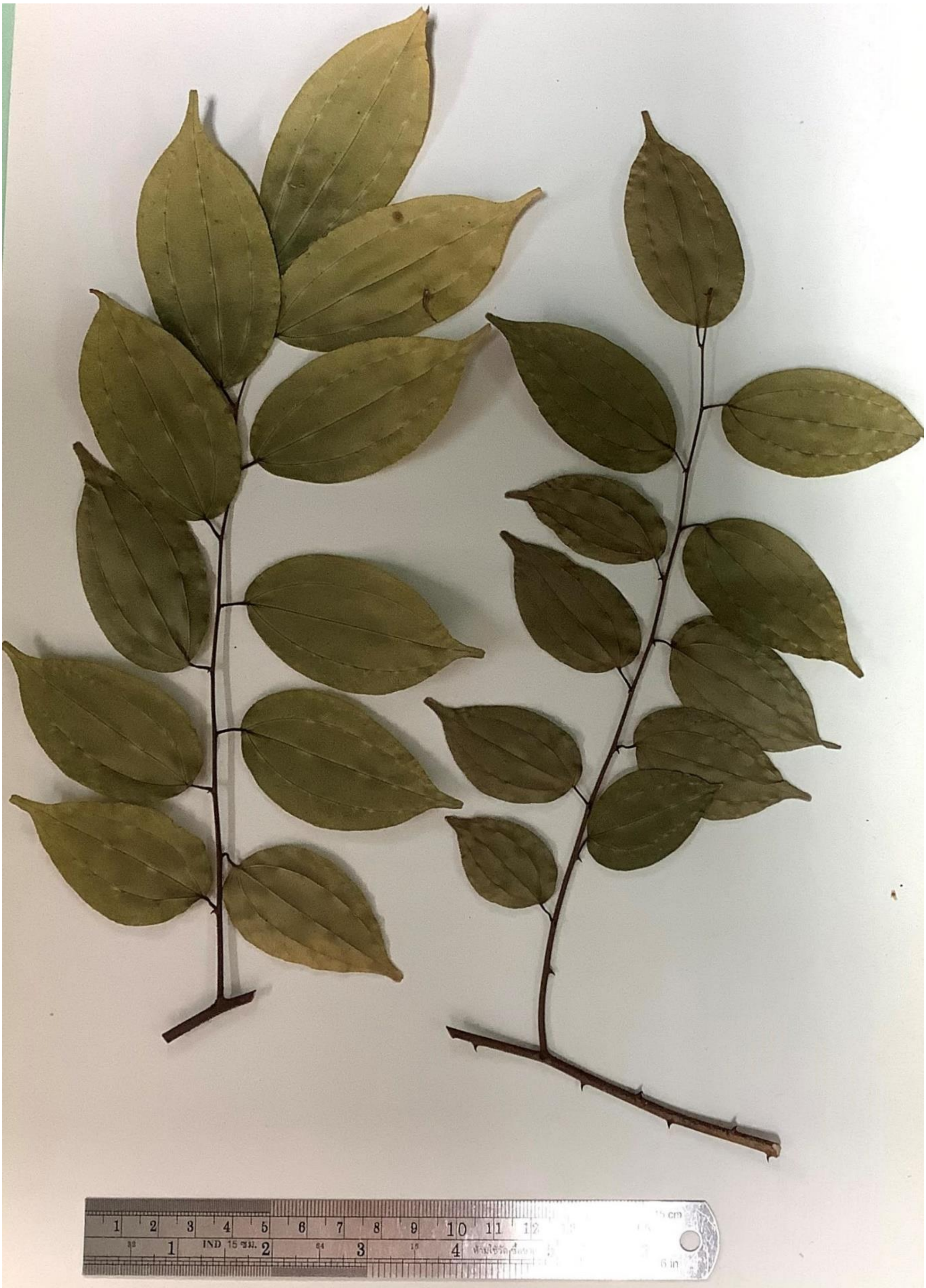

S1 Fig XZ. ZA3 specimen.
