# Supplementary material for: Verification of Thai ethnobotanical medicine “Kamlang Suea Khrong” driven by multiplex PCR and powerful TLC techniques
Source: PLoS One. 2021 Sep 17;16(9):e0257243. doi: 10.1371/journal.pone.0257243 (PMC8448358; doi:10.1371/journal.pone.0257243)
Supplement: S2 Appendix — (PDF) [file pone.0257243.s005.pdf]

S2 Appendix. Input data for HCA cluster analysis.

S2 Table A. Input data from TLC chromatograms of the authentic samples.

Sample code	Rf 1.7	Rf 2.0	Rf 2.3	Rf 2.7	Rf 4.1	Rf 4.4	Rf 6.9
**BA1**	0	1	1	1	0	0	1
**BA2**	0	0	1	1	0	0	0
**BA3**	0	0	1	1	0	0	1
**BA4**	0	0	1	1	0	0	0
**BA5**	0	0	0	1	0	0	0
**SA1**	0	1	0	0	1	0	1
**SA2**	0	1	0	0	1	0	1
**ZA1**	1	1	1	1	1	1	1
**ZA2**	1	1	1	1	1	1	1

S2 Table B. Input data from TLC chromatograms of the authentic samples (BA1, SA1, and ZA1) and commercial KSK crude drugs (CK1-CK5).

Sample code	Rf 2.5	Rf 3.0	Rf 4.2	Rf 6.0-6.2	Rf 7.0
**BA1**	0	1	0	1	1
**SA1**	0	0	1	0	1
**ZA1**	1	1	1	1	1
**CK1**	0	1	0	1	1
**CK2**	0	1	0	1	1
**CK3**	1	1	1	1	0
**CK4**	1	1	1	1	0
**CK5**	1	1	1	1	1
