# Supplementary material for: Verification of Thai ethnobotanical medicine “Kamlang Suea Khrong” driven by multiplex PCR and powerful TLC techniques
Source: PLoS One. 2021 Sep 17;16(9):e0257243. doi: 10.1371/journal.pone.0257243 (PMC8448358; doi:10.1371/journal.pone.0257243)
Supplement: S3 Appendix — (PDF) [file pone.0257243.s006.pdf]

S3 Appendix.

Variation in ITS region of *S. axillaris* and *Z. attopensis*.

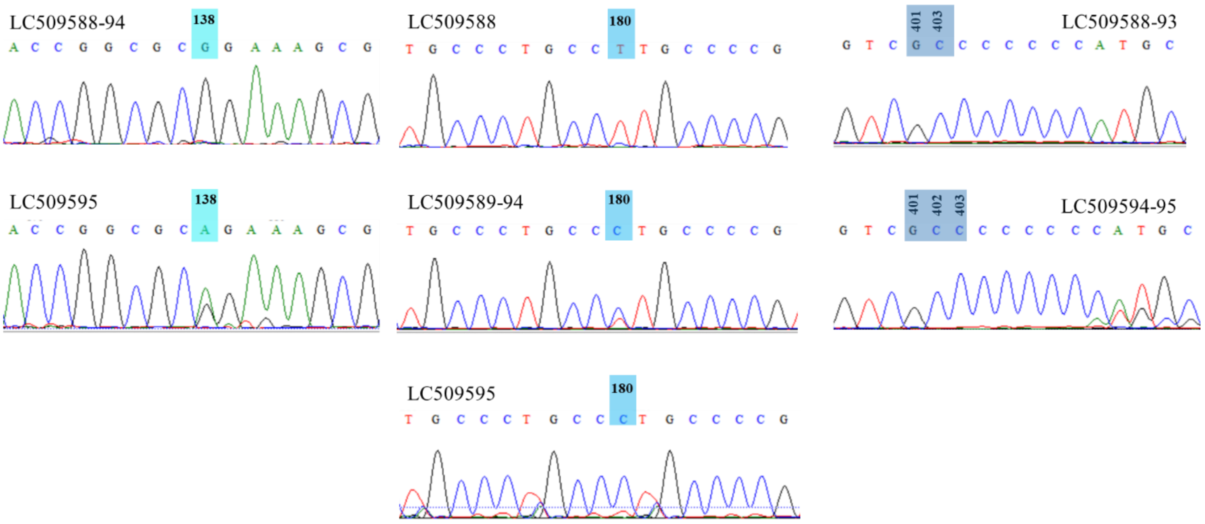

S3 Fig A. SNPs in the ITS region amplified from leaves of *S. axillaris*.

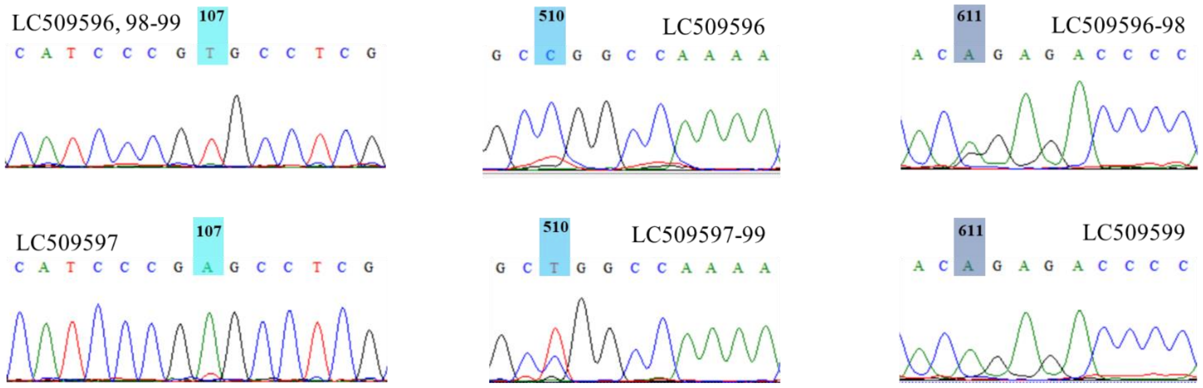

S3 Fig B. SNPs in the ITS region amplified from leaves of *Z. attopensis*.
